# Carbon footprint of biomass-derived activated carbon: life cycle assessment, environmental sustainability, and carbon-negative potential

**DOI:** 10.1039/d6ra06867g

**Published:** 2026-10-19

**Authors:** Ikhazuagbe Hilary Ifijen

**Affiliations:** a Department of Chemistry, Edo State University, Iyamho, Uzairue Edo State Nigeria larylans4u@yahoo.com

## Abstract

Biomass-derived activated carbon has emerged as a sustainable alternative to conventional fossil-based activated carbon owing to its renewable origin, excellent adsorption performance, and potential to support circular bioeconomy and climate mitigation strategies. However, its overall environmental sustainability depends not only on the renewable nature of biomass feedstocks but also on greenhouse gas emissions generated throughout production, utilization, regeneration, and end-of-life management. This review critically reviews the carbon footprint of biomass-derived activated carbon from a life cycle perspective, emphasizing the influence of feedstock selection, production technologies, activation methods, transportation, energy consumption, and chemical utilization on environmental performance. The application of life cycle assessment (LCA) and carbon accounting frameworks for quantifying greenhouse gas emissions, identifying environmental hotspots, and evaluating carbon-negative potential is comprehensively discussed. Comparative analyses of diverse biomass feedstocks and recent experimental studies demonstrate that agricultural residues generally exhibit lower environmental burdens than fossil-derived precursors, although sustainability remains highly dependent on process design, regional energy systems, and methodological assumptions. Emerging applications in water purification, air pollution control, carbon dioxide capture, catalysis, energy storage, hydrogen storage, and soil carbon sequestration further illustrate the multifunctional role of biomass-derived activated carbon in sustainable development. Finally, current challenges, knowledge gaps, and future research priorities are highlighted to facilitate the development of low-carbon, resource-efficient, and industrially scalable activated carbon production systems.

## Abstract 2

Biomass-based activated carbon presents a sustainable, high quality substitute for fossil-based carbon, in the framework of a circular bioeconomy and climate change mitigation.

## Graphical abstract

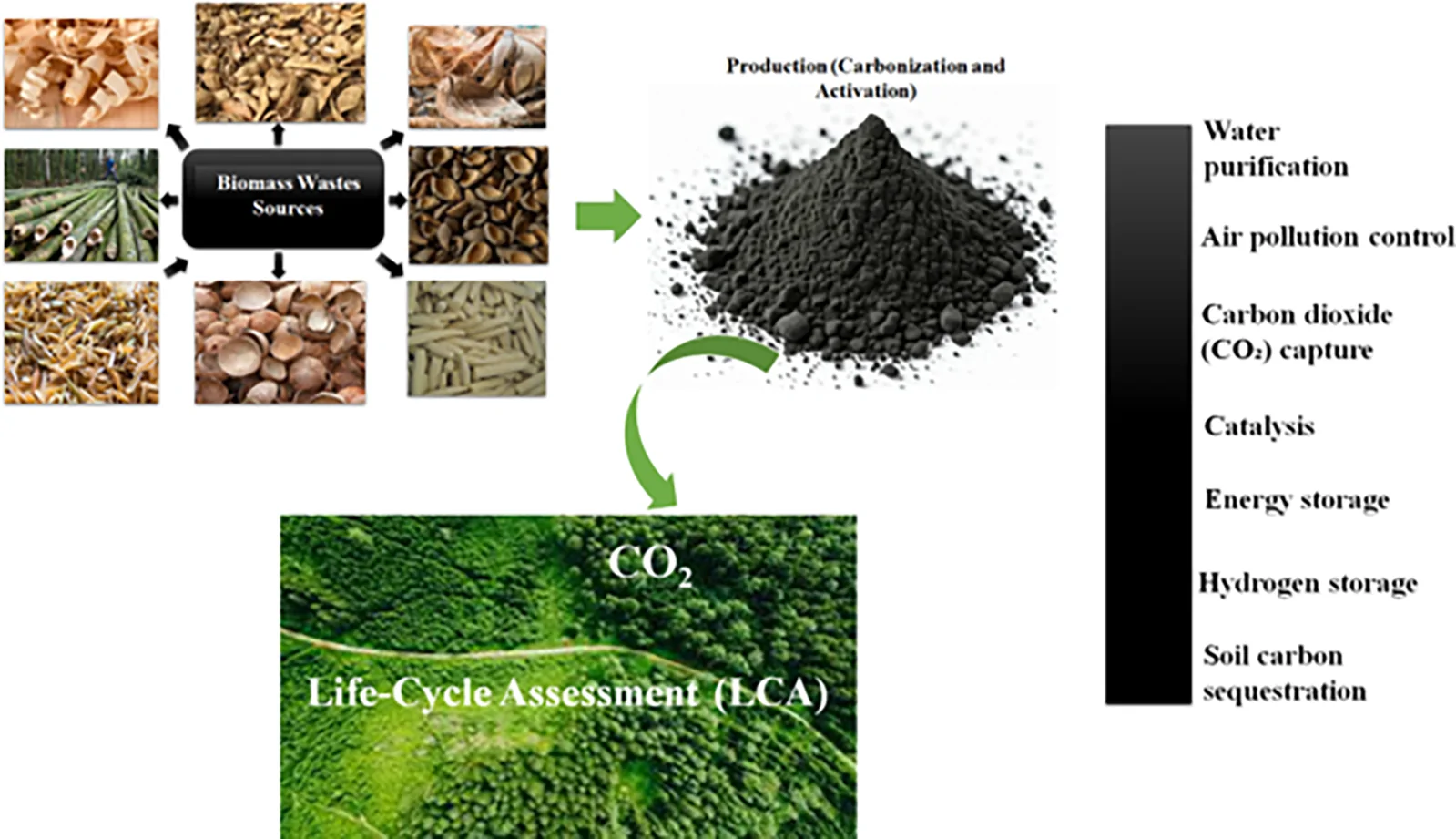

## 1 Introduction

Climate change remains one of the greatest environmental challenges confronting modern society, driven primarily by the continuous increase in anthropogenic greenhouse gas (GHG) emissions associated with fossil fuel combustion, industrialization, transportation, land-use change, and inefficient waste management.^1–3^ The accumulation of atmospheric carbon dioxide (CO_2_), the dominant greenhouse gas, has intensified global warming, contributing to rising sea levels, biodiversity loss, ecosystem degradation, and an increased frequency of extreme climatic events. These consequences have reinforced the urgency of developing sustainable materials and technologies capable of reducing GHG emissions while promoting efficient resource utilization. Consequently, global initiatives aimed at achieving the targets of the Paris Agreement have accelerated the transition toward low-carbon manufacturing systems and environmentally sustainable production practices.^4–6^

In recent years, the concepts of net-zero emissions and carbon-negative technologies have become central to international climate mitigation strategies. Net-zero systems seek to balance greenhouse gas emissions with equivalent carbon removals, whereas carbon-negative systems require net removal of atmospheric carbon after accounting for attributable emissions across the defined system boundary.^7,8^ Achieving these goals requires greater emphasis on renewable feedstocks, circular resource utilization, and waste valorization as potential pathways for reducing environmental impacts while creating economic value.^9,10^

Among the diverse classes of sustainable materials, biomass-derived activated carbon has emerged as a particularly attractive carbonaceous material because it combines renewable resource utilization with high-value functional performance. Activated carbon possesses an exceptionally high specific surface area, well-developed pore structure, and abundant surface functional groups, making it one of the most versatile adsorbent materials for environmental and industrial applications. It has been widely employed in water and wastewater treatment, air purification, gas separation, catalysis, energy storage, and carbon capture owing to its excellent adsorption capacity and chemical stability. Traditionally, activated carbon has been manufactured from fossil-derived precursors such as coal and petroleum coke; however, growing environmental concerns and increasing demand for sustainable materials have stimulated the use of renewable biomass feedstocks, including agricultural residues, forestry wastes, food-processing by-products, municipal organic waste, and animal-derived biomass. Converting these resources into activated carbon can reduce dependence on non-renewable raw materials while supporting waste minimization, resource recovery, and circular-economy principles.^11–13^

Despite these advantages, the environmental sustainability of biomass-derived activated carbon cannot be evaluated solely on the basis of renewable feedstock utilization. Its production involves multiple stages—including feedstock collection, transportation, pretreatment, carbonization, activation, purification, drying, and product distribution—each of which can require energy, chemicals, and water. Moreover, production technologies, activation methods, feedstock characteristics, and energy sources differ considerably among manufacturing systems, resulting in substantial variations in GHG emissions and resource consumption.^12,13^ Consequently, a comprehensive assessment is required to determine whether biomass-derived activated carbon provides environmental benefits compared with conventional fossil-based alternatives. Importantly, a lower carbon footprint relative to fossil-derived activated carbon does not, by itself, demonstrate carbon-negative performance; such a claim requires consideration of life-cycle emissions together with the quantity, stability, and duration of carbon retained or removed through the production and subsequent life cycle of the material.

Life cycle assessment (LCA) provides an established framework for evaluating these environmental impacts. Standardized under the ISO 14040 and ISO 14044 series, LCA assesses the environmental performance of products throughout their defined life cycle, from raw material acquisition through production, use, regeneration, and end-of-life management. Beyond GHG emissions, LCA can evaluate multiple environmental indicators, including global warming potential, cumulative energy demand, water consumption, acidification, eutrophication, and resource depletion. Such assessment enables identification of environmental hotspots and comparison of alternative production pathways. However, reported environmental performance can vary substantially according to system boundaries, functional units, treatment of biogenic carbon, energy sources, feedstock allocation, transportation assumptions, and credits assigned to avoided emissions or carbon retention.^17–20^ These methodological differences can therefore complicate direct comparison among studies and influence the interpretation of biomass-derived activated-carbon pathways.

This methodological complexity, together with the need to distinguish lower-carbon performance from demonstrable carbon negativity, provides the basis for the present comprehensive and critical review. The review integrates evidence on carbon-footprint assessment, LCA methodology, biomass waste valorization, process-level emission hotspots, carbon retention, avoided emissions, regeneration, and end-of-life carbon fate to critically evaluate the conditions under which biomass-derived activated carbon may achieve a net-negative carbon balance. Table 1 shows the comparison of existing review studies and positioning of the present review.

**Table 1:** Comparison of existing review studies and positioning of the present review

Review study	Main focus	LCA/carbon footprint	Carbon-negative potential	Key limitation
Bedia *et al.* (2020)^18^	Activated-carbon preparation and chemical activation	Limited	Not addressed	Focuses mainly on activation and material preparation
Wang *et al.* (2021)^19^	Activated-carbon modification and adsorption	Not addressed	Not addressed	Emphasizes material properties and adsorption performance
Sharma *et al.* (2022)^21^	Sustainable and multifunctional activated carbon	Limited	Not addressed	Sustainability discussed broadly without detailed carbon accounting
Serafin & Dziejarski (2023)^22^	Activated-carbon preparation and CO_2_ capture	Limited	Limited	Focuses mainly on preparation and CO_2_-capture applications
Hossen *et al.* (2026)^11^	Biomass-derived activated carbon and sustainable applications	Limited	Limited	Broad sustainability perspective without an integrated carbon-negative framework
Villora-Picó *et al.* (2024)^12^	Renewable biomass-derived carbon materials	Limited	Not addressed	Broader carbonaceous-material scope rather than activated-carbon carbon accounting
Manimekala *et al.* (2025)^13^	Biomass-derived activated carbon for supercapacitors	Limited	Not addressed	Primarily focused on energy-storage applications
Present review	Carbon footprint, LCA, sustainability and carbon-negative potential of biomass-derived activated carbon	Comprehensive	Comprehensive	Integrates production, emissions, carbon retention, avoided emissions, use, regeneration and end-of-life carbon fate

Accordingly, the review examines the fundamental characteristics of biomass-derived activated carbon, major carbon-emission sources throughout its production chain, and the application of LCA for evaluating environmental performance. Particular attention is given to how feedstock selection, transportation, pretreatment, carbonization, activation technology, process energy, chemical consumption, product performance, regeneration, and end-of-life management influence the overall life-cycle carbon balance. The review also synthesizes evidence on environmental sustainability, emerging applications, industrial-scale implementation, current challenges, and future research needs.

Rather than assuming that biomass-derived activated carbon is inherently carbon negative, this review critically evaluates the evidence and methodological conditions required to substantiate such a claim. By linking biomass valorization and material functionality with life-cycle carbon accounting, the review aims to identify the factors that determine whether biomass-derived activated carbon represents merely a renewable or lower-carbon alternative or can be credibly classified as a carbon-negative material.

## 2 Literature search and critical review methodology

This review employed a structured literature-search and critical-synthesis approach to identify and evaluate relevant evidence across biomass conversion, activated-carbon production, adsorption, environmental assessment, life-cycle assessment (LCA), process engineering, and sustainability research. The literature was screened and synthesized according to the predefined search and selection criteria described below, with particular attention to studies reporting environmental performance, carbon emissions, LCA results, regeneration, scale-up, techno-economic considerations, and carbon-negative potential of biomass-derived activated carbon.

The literature was reviewed using a structured search and thematic synthesis approach. The review was not intended as a formal systematic review or meta-analysis because the available studies differ substantially in feedstock characteristics, preparation routes, activation conditions, adsorption applications, experimental scales, LCA system boundaries, functional units, energy systems, and carbon-accounting assumptions. A critical-review approach was therefore considered more appropriate for integrating the heterogeneous evidence and evaluating areas of agreement, uncertainty, methodological inconsistency, and research gaps.

### 2.1 Literature search strategy

The literature search focused on peer-reviewed publications addressing biomass-derived activated carbon and related carbonaceous materials, with emphasis on studies relevant to production, adsorption performance, environmental sustainability, carbon footprint, and carbon-negative potential. Searches were structured around combinations of keywords relating to biomass and agricultural residues, activated carbon, carbonization, pyrolysis, physical and chemical activation, CO_2_ adsorption, regeneration, carbon footprint, life-cycle assessment, environmental impact, techno-economic analysis, scale-up, carbon sequestration, carbon retention, and carbon-negative technologies.

Representative search combinations included:

(Biomass OR agricultural waste OR lignocellulosic biomass OR biomass residue) AND (activated carbon OR biomass-derived activated carbon OR biochar-derived carbon) AND (CO_2_ capture OR carbon dioxide adsorption OR gas adsorption) AND (carbon footprint OR life-cycle assessment OR LCA OR environmental impact OR carbon-negative) AND (activation OR pyrolysis OR carbonization OR regeneration OR scale-up OR techno-economic analysis).

Additional targeted searches were undertaken where specific evidence was required for individual sections of the review. These searches considered individual biomass feedstocks, activation technologies, regeneration methods, environmental indicators, process-energy requirements, industrial-scale production, and techno-economic performance. Relevant references cited by key primary studies and review articles were also examined to identify additional publications and complementary evidence.

### 2.2 Literature selection and scope

The literature was selected according to its relevance to the central objectives and thematic scope of the review. Particular attention was given to studies providing evidence on one or more of the following areas: (i) biomass feedstock characteristics, availability, and logistics; (ii) pretreatment, carbonization, and activation; (iii) physicochemical characteristics and adsorption performance of activated carbon; (iv) process-energy and chemical requirements; (v) regeneration and repeated-use performance; (vi) life-cycle assessment and carbon-footprint quantification; (vii) environmental impacts and carbon retention; (viii) industrial-scale production and process integration; (ix) techno-economic assessment; and (x) carbon-negative or carbon-removal potential.

Priority was given to peer-reviewed experimental studies, LCA studies, process-modelling and process-design studies, techno-economic assessments, pilot- and industrial-scale investigations, and relevant review articles that provided sufficient methodological or quantitative information for critical evaluation. Studies were not considered central evidence where they were outside the scope of biomass-derived activated carbon or provided insufficient information to support the technological, environmental, economic, or carbon-balance issues examined in the review.

The evidence base was not restricted to a single application because the environmental performance of activated carbon depends on the complete production and utilization pathway rather than on adsorption capacity alone. Accordingly, studies involving CO_2_ capture and other adsorption applications were considered where they provided transferable information on feedstock selection, activation, energy demand, regeneration, environmental performance, or carbon retention.

### 2.3 Thematic organization and critical synthesis

The selected literature was organized thematically according to the major stages and determinants of the biomass-derived activated-carbon life cycle. These included feedstock selection and sourcing, transportation, pretreatment, carbonization, activation, process energy, chemical consumption, adsorption performance, regeneration, end-of-life management, environmental assessment, industrial-scale implementation, and techno-economic feasibility.

The review goes beyond a descriptive summary by comparing findings across studies and examining the factors responsible for differences in reported performance. Particular attention was given to feedstock composition, moisture content, transportation distance, carbon yield, activation severity, activating-agent consumption, energy source, product yield, adsorption capacity, regeneration requirements, and carbon retention. This approach allows technical performance to be considered alongside the environmental burdens associated with producing and using the adsorbent.

For studies involving LCA or carbon-footprint assessment, reported results were interpreted in relation to system boundaries, functional units, allocation procedures, electricity mixes, transportation assumptions, treatment of coproducts, and accounting of biogenic carbon. Such methodological differences can substantially influence the magnitude and even the direction of reported environmental benefits. Consequently, numerical results from individual studies were not treated as universally comparable values; instead, they were interpreted within the methodological context of each assessment.

### 2.4 Critical assessment of carbon footprint and carbon-negative claims

A central objective of the review was to distinguish between the concepts of renewable feedstock use, reduced carbon footprint, carbon neutrality, carbon retention, and genuinely carbon-negative performance. The renewable origin of biomass was therefore not considered sufficient evidence of a carbon-negative product. Claims of carbon negativity were critically examined in relation to the complete life-cycle carbon balance, including biomass acquisition and transportation, pretreatment, carbonization, activation, process energy, chemical inputs, emissions, avoided emissions, adsorbent utilization, regeneration, and end-of-life carbon fate.

Particular emphasis was placed on whether studies adequately considered the source of process energy, upstream emissions associated with activating chemicals and electricity, carbon retained in the final activated carbon, treatment of coproducts, and the fate of carbon following use and disposal. This distinction is essential because a biomass-derived material may have a lower carbon footprint than a fossil-derived alternative without necessarily achieving a net negative carbon balance.

### 2.5 Evaluation of technological, environmental, and economic evidence

Technical, environmental, and economic evidence was evaluated together because improvements in material performance do not necessarily translate into improved life-cycle sustainability or commercial viability. Studies reporting high surface area or adsorption capacity were therefore considered alongside adsorbent yield, activation intensity, energy consumption, chemical requirements, regeneration performance, and process scale.

Similarly, techno-economic evidence was interpreted in relation to feedstock availability and cost, transportation, plant capacity, energy consumption, chemical requirements, product yield, process configuration, and potential product value. Where quantitative economic results were available, they were used as benchmarks within the conditions and assumptions of the individual studies rather than generalized to all biomass feedstocks and production technologies.

Industrial-scale evidence was also distinguished from laboratory-scale evidence. Pilot-scale operation, industrial process design, and demonstrated commercial integration were treated as different levels of technological maturity. This distinction prevents laboratory adsorption performance or modelled process feasibility from being interpreted as evidence of established commercial deployment.

### 2.6 Evidence limitations and rationale for the review approach

The literature on biomass-derived activated carbon is highly heterogeneous. Studies vary considerably in biomass type, preparation route, activation technology, operating conditions, scale, analytical methods, LCA boundaries, functional units, energy systems, and carbon-accounting approaches. These differences limit the validity of direct numerical comparison and make statistical pooling inappropriate for the present review.

Accordingly, the evidence was synthesized qualitatively and comparatively, with emphasis on identifying consistent trends, process trade-offs, environmental hotspots, economic constraints, methodological uncertainties, and research gaps. Where apparently conflicting findings were encountered, differences in feedstock characteristics, process conditions, system boundaries, or methodological assumptions were considered before drawing conclusions.

This methodology supports the central purpose of the review: to provide a comprehensive assessment of biomass-derived activated carbon while critically examining whether its environmental advantages extend beyond the use of renewable feedstocks to encompass the full production and utilization life cycle. The approach therefore provides a basis for evaluating when biomass-derived activated carbon can be considered a lower-carbon material and, under sufficiently favourable process and carbon-accounting conditions, when it may credibly contribute to carbon-negative material systems.

## 3 Biomass-derived activated carbon

### 3.1 Fundamentals of activated carbon

Activated carbon is a highly porous carbonaceous material distinguished by its exceptionally large internal surface area, well-developed pore network, and chemically active surface. These characteristics collectively confer outstanding adsorption properties, making activated carbon one of the most versatile materials for environmental remediation, industrial purification, catalysis, and energy-related applications. Although activated carbon can be produced from a wide variety of carbonaceous precursors, biomass-derived activated carbon has attracted increasing attention because it combines high adsorption performance with renewable feedstocks, waste valorization, and reduced reliance on fossil resources. The intrinsic physicochemical properties of activated carbon are governed primarily by its pore architecture and surface chemistry, both of which are strongly influenced by the nature of the precursor material and the activation process. However, the environmental advantages of biomass-derived activated carbon cannot be inferred solely from its renewable origin; life-cycle studies have demonstrated that feedstock choice, activation route, energy requirements, transportation, and the treatment of biogenic carbon can substantially influence its overall carbon footprint and environmental performance.^17,18^Table 2 shows the fundamental physicochemical characteristics governing the adsorption performance of activated carbon.^17,18^

**Table 2:** Fundamental physicochemical characteristics governing the adsorption performance of activated carbon^24–30^

Property	Description	Functional significance
Adsorption mechanism	Adsorption occurs through physical (van der Waals forces) and/or chemical interactions between the adsorbent surface and adsorbate molecules	Determines adsorption capacity, selectivity, and regeneration efficiency
Specific surface area	Typically ranges from 500–2500 m^2^ g^−1^ depending on precursor and activation method	Provides abundant active sites for adsorption and catalytic processes
Micropores (<2 nm)	Small pores responsible for most of the internal surface area	Predominantly responsible for adsorption of gases and low-molecular-weight contaminants
Mesopores (2–50 nm)	Intermediate pores connecting the external surface with micropores	Facilitate diffusion and adsorption of larger organic molecules, improving adsorption kinetics
Macropores (>50 nm)	Large transport pores that provide access to the internal pore network	Enhance mass transfer and reduce diffusion resistance
Surface functional groups	Oxygen-containing groups (hydroxyl, carboxyl, carbonyl, phenolic) and, in some cases, nitrogen- or sulfur-containing functionalities	Regulate surface polarity, acid–base behavior, hydrophilicity, and adsorption selectivity
Surface chemistry	Combination of graphitic carbon domains and chemically active surface functionalities	Influences electrostatic interactions, hydrogen bonding, π–π interactions, and redox activity
Hierarchical pore structure	Integrated network of macro-, meso-, and micropores	Maximizes adsorption capacity by improving accessibility to internal adsorption sites

Adsorption is the fundamental mechanism responsible for the functionality of activated carbon. Unlike absorption, where molecules penetrate the bulk of a material, adsorption involves the accumulation of atoms, ions, or molecules on the carbon surface through physical or chemical interactions. Physical adsorption is predominantly governed by van der Waals forces and is generally reversible, making activated carbon highly suitable for applications requiring adsorbent regeneration. In contrast, chemical adsorption involves stronger interactions through covalent or ionic bonding between surface functional groups and adsorbate molecules, often resulting in greater selectivity but lower reversibility. In practical systems, both mechanisms may operate simultaneously, with their relative contributions depending on the physicochemical characteristics of the adsorbent and the adsorbate, as well as operating conditions such as temperature, pH, and solution chemistry.^19,20^

The remarkable adsorption capacity of activated carbon arises primarily from its highly developed porous structure, which provides a large accessible internal surface area for adsorbate–surface interactions. The pore network is generally classified into three categories according to pore diameter: micropores (<2 nm), mesopores (2–50 nm), and macropores (>50 nm). Each pore class contributes differently to the adsorption process. Micropores provide a major proportion of the internal surface area and are particularly important for the adsorption of small molecules, gases, and dissolved contaminants. Their narrow dimensions generate strong adsorption potentials, thereby promoting efficient adsorption through pore-filling and related confinement mechanisms.^20–23^

Mesopores serve a complementary function by facilitating the transport of adsorbates into the interior microporous network. Their larger dimensions can reduce mass-transfer limitations and enhance diffusion, particularly for relatively large organic molecules such as dyes, pharmaceuticals, natural organic matter, and other complex pollutants. Consequently, activated carbons with an appropriate balance of micropores and mesopores can exhibit improved adsorption kinetics and broader applicability across environmental and industrial processes. Macropores generally contribute less to the total surface area but function predominantly as transport pathways that facilitate fluid penetration and provide access to smaller adsorption pores. Thus, the hierarchical integration of macro-, meso-, and micropores can enhance adsorption capacity and kinetics by improving molecular transport throughout the porous structure.^24,25^

Equally important to adsorption performance is the surface chemistry of activated carbon. The carbon surface contains numerous oxygen-containing functional groups, including hydroxyl, carbonyl, carboxyl, phenolic, lactonic, and quinone groups, together with smaller quantities of nitrogen-, sulfur-, or phosphorus-containing functionalities depending on the precursor material and activation conditions. These surface functionalities influence hydrophilicity, surface charge, acid–base characteristics, electron transfer behavior, and adsorption selectivity. Oxygen-containing functional groups generally enhance interactions with polar compounds through hydrogen bonding and electrostatic attraction, whereas graphitic carbon domains favor the adsorption of nonpolar organic molecules *via* π–π interactions and hydrophobic forces. Consequently, the adsorption behavior of activated carbon depends not only on its pore characteristics but also on the chemical composition and distribution of surface functional groups.^26,27^

The physicochemical properties of biomass-derived activated carbon are closely linked to the composition of the original biomass precursor. Lignocellulosic materials composed of cellulose, hemicellulose, and lignin undergo complex thermal decomposition during carbonization, generating a carbon-rich framework that is subsequently modified during activation to produce the characteristic porous structure. Variations in biomass composition, ash content, mineral constituents, and inherent microstructure therefore influence pore development, surface chemistry, carbon yield, and adsorption performance. Furthermore, activation conditions—including temperature, residence time, activating agent, and activation atmosphere—can be tailored to regulate pore size distribution and surface functionality, enabling the production of activated carbons with properties optimized for specific applications.^27,28^

The unique combination of high surface area, hierarchical porosity, tunable surface chemistry, and renewable origin has established biomass-derived activated carbon as a versatile functional material capable of addressing diverse environmental and technological challenges. These fundamental characteristics form the scientific basis for understanding how biomass feedstocks, activation technologies, and life cycle considerations collectively influence the performance and sustainability of activated carbon production, topics discussed in the subsequent sections.

### 3.2 Biomass precursor selection and its influence on activated carbon performance

The selection of biomass precursor is a fundamental determinant of the physicochemical characteristics, production efficiency, and environmental sustainability of activated carbon. Unlike conventional coal- and petroleum-derived precursors, biomass feedstocks are renewable, inexpensive, and abundantly available as agricultural, industrial, and municipal residues. Their utilization not only diverts substantial quantities of organic waste from landfills but also aligns with the principles of waste valorization and the circular bioeconomy by transforming low-value biomass into high-value porous carbon materials.^6,7^ However, biomass feedstocks differ considerably in their chemical composition, particularly in cellulose, hemicellulose, lignin, extractives, ash, and mineral contents, resulting in significant variations in carbon yield, pore development, surface chemistry, mechanical stability, and adsorption performance. Consequently, biomass selection should not be based solely on availability but rather on the intrinsic properties required for the intended application of the activated carbon. The principal physicochemical characteristics governing the adsorption performance of activated carbon and their functional significance are summarized in Table 3.

**Table 3:** Representative biomass feedstocks used for activated carbon production^4,6,7,31–34^

Biomass category	Representative feedstocks	Major constituents	Typical characteristics	Representative applications
Agricultural residues	Rice husk, coconut shell, corn cob, sawdust	Cellulose, hemicellulose, lignin	High carbon yield, well-developed porosity, renewable and abundant	Water treatment, gas adsorption, catalysis, energy storage
Animal-derived biomass	Animal bones, shells	Organic carbon, calcium phosphate, calcium carbonate	Mineral-rich structure, enhanced surface functionality, waste valorization	Heavy metal adsorption, fluoride removal, catalysis
Industrial/municipal wastes	Sewage sludge, food waste	Organic matter, minerals, ash	Variable composition, supports circular economy, large waste volumes	Wastewater treatment, pollutant removal, soil remediation

Biomass feedstocks employed for activated carbon production can generally be categorized into agricultural residues, animal-derived biomass, and industrial or municipal organic wastes. Although each category offers unique advantages, no single precursor is universally superior. Instead, the suitability of a biomass depends on its chemical composition, activation behavior, economic viability, and environmental implications.^8,23,24^ The major categories of renewable biomass feedstocks used for activated carbon production are illustrated in Fig. 1, highlighting the diversity of precursor materials available for carbonization and activation. This diversity, however, creates an important selection problem: a precursor that performs well in terms of adsorption capacity may not necessarily provide the best carbon yield, regeneration stability, process efficiency, or life-cycle environmental performance. Thus, precursor selection represents a trade-off between material performance and the environmental and economic burdens associated with converting the biomass into activated carbon.

**Fig. 1:**
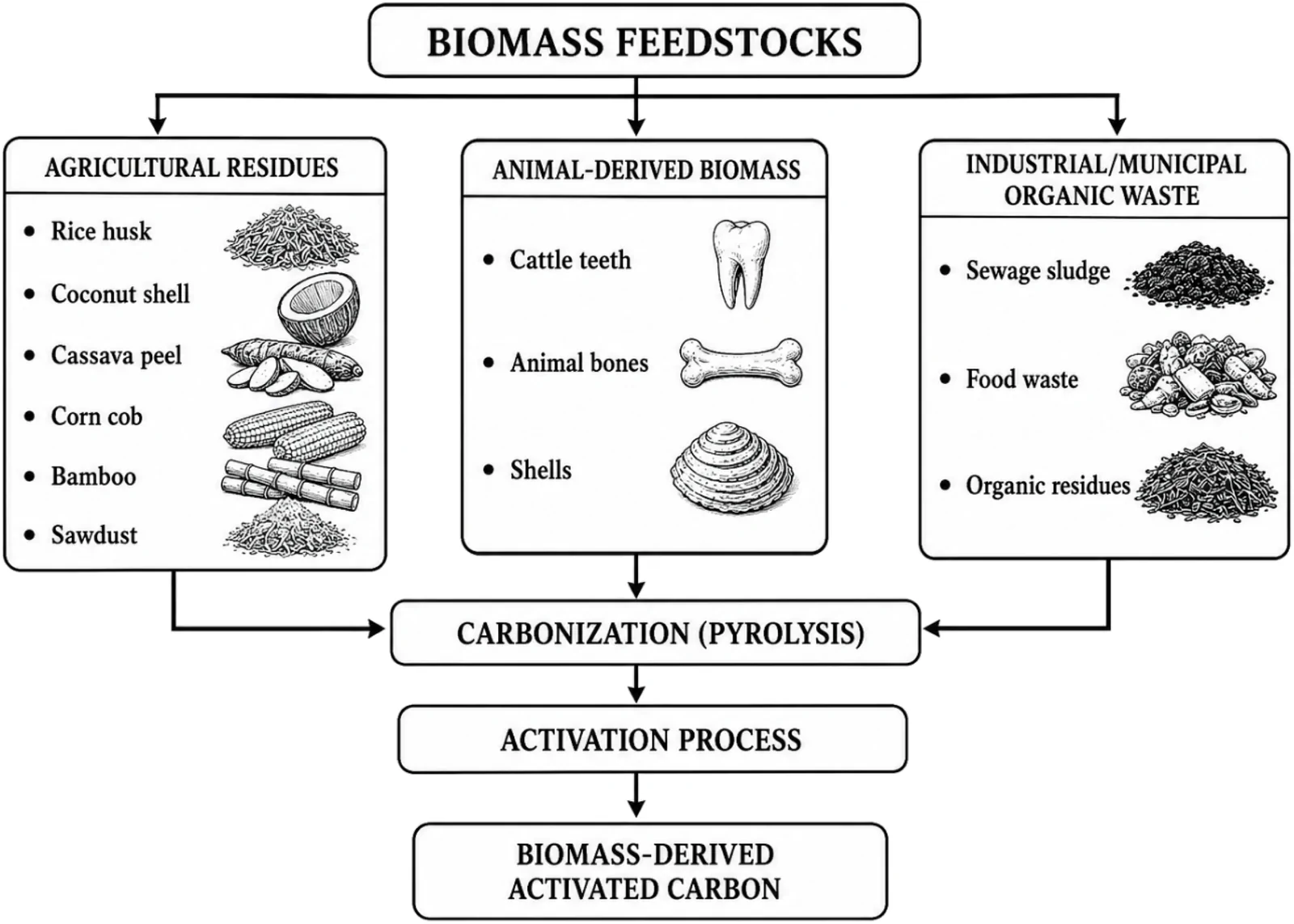
Classification of the principal biomass feedstocks used for activated carbon production. Agricultural residues, animal-derived biomass, and industrial or municipal organic wastes represent the major renewable precursor categories that can be transformed into high-value activated carbon through carbonization and activation processes.

Agricultural residues remain the most extensively investigated precursors because of their widespread availability, renewability, and favorable lignocellulosic composition. Feedstocks such as coconut shells, rice husks, cassava peels, bamboo, corn cobs, and sawdust have consistently demonstrated potential for producing activated carbons with well-developed pore structures and high adsorption capacities. Nevertheless, substantial differences exist among these materials. Coconut shells and bamboo, which possess relatively high lignin contents, generally produce activated carbons with greater mechanical strength, higher carbon yields, and superior structural integrity than cellulose-rich agricultural residues. In contrast, rice husks contain appreciable quantities of silica, which influences pore development and ash content and may either improve structural rigidity or reduce adsorption efficiency depending on the activation strategy employed. Consequently, the physicochemical composition of the precursor exerts a greater influence on activated carbon quality than biomass abundance alone.^6–8^ This observation challenges the common assumption that the most abundant or readily available agricultural residue is necessarily the most suitable precursor. Rather, differences in lignin, mineral, and ash content can determine how efficiently the precursor is converted into a functional porous structure, meaning that feedstock chemistry must be considered alongside local availability and intended application.

The superiority of lignin-rich agricultural residues has been demonstrated experimentally. Badiger and Nidheesh (2024)^6^ produced activated biochar from coconut shell possessing a BET surface area of 368.07 m^2^ g^−1^ with well-developed micro- and macroporous structures confirmed by X-ray microtomography and scanning electron microscopy. Surface characterization further revealed abundant oxygen-containing functional groups and aromatic carbon structures that promoted the adsorption of acetaminophen and ciprofloxacin through chemisorption mechanisms best described by pseudo-second-order kinetics and the Freundlich isotherm. Despite reduced adsorption efficiency under competitive adsorption conditions, the material maintained high ciprofloxacin removal efficiency after repeated regeneration cycles, highlighting the durability and reusability of coconut shell-derived activated carbon for water purification. Importantly, these results also demonstrate why high initial adsorption capacity should not be treated as a sufficient indicator of precursor superiority. The decline in performance under competitive conditions indicates that adsorption behavior depends on the chemical complexity of the treatment matrix, while regeneration performance provides an additional criterion for evaluating practical sustainability. These findings reinforce the importance of precursor composition in developing activated carbons with both favorable pore architecture and surface functionality suitable for environmental remediation.

Animal-derived biomass has recently emerged as an attractive alternative precursor because its adsorption behavior is governed not only by carbonaceous pore structures but also by naturally occurring mineral constituents such as calcium phosphate and calcium carbonate. Feedstocks including cattle bones, animal teeth, fish bones, and mollusk shells therefore possess adsorption mechanisms that extend beyond conventional pore filling to include ion exchange, surface complexation, and mineral-assisted precipitation. These additional mechanisms make mineral-rich biomass particularly attractive for the removal of heavy metals, phosphate, and fluoride from aqueous environments. However, their relatively high inorganic and ash contents may reduce carbon yield and specific surface area compared with lignocellulosic biomass, indicating that the choice between agricultural and animal-derived feedstocks should ultimately depend on the target contaminant and desired adsorption mechanism rather than surface area alone. This distinction is particularly important for sustainable process design because maximizing BET surface area may be counterproductive when the dominant contaminant-removal mechanism is mineral-mediated rather than pore-driven. Accordingly, precursor evaluation should consider the mechanism by which adsorption occurs, rather than ranking materials solely according to conventional surface-area metrics.

Industrial and municipal organic wastes have attracted increasing attention because they simultaneously address waste management challenges and provide sustainable carbon precursors. Sewage sludge, food waste, and biodegradable industrial residues represent abundant yet underutilized resources capable of producing activated carbons suitable for environmental applications. Nevertheless, these feedstocks exhibit greater compositional heterogeneity than agricultural biomass, often containing significant quantities of inorganic minerals, heavy metals, and other contaminants that may adversely affect product quality unless appropriate pretreatment and activation strategies are employed. Consequently, optimizing processing conditions remains essential for transforming these heterogeneous wastes into high-performance activated carbons. The principal advantage of these feedstocks is therefore not necessarily superior intrinsic adsorption performance, but their potential to integrate waste treatment with material production. At the same time, this advantage can be undermined if pretreatment, contaminant removal, or energy-intensive activation introduces additional environmental burdens. Their sustainability must therefore be demonstrated rather than assumed from their waste origin.

Recent experimental evidence demonstrates that these challenges can be successfully overcome. Aljubiri *et al.* (2024)^7^ synthesized sludge-based activated carbons from thickened and unthickened sewage sludge with BET surface areas of 336.34 and 498.39 m^2^ g^−1^, respectively. Both materials exhibited exceptional adsorption performance, removing approximately 99.99% of Cd^2+^ and Pb^2+^ from aqueous solutions while maintaining excellent regeneration performance over five adsorption–desorption cycles. The adsorption process followed pseudo-second-order kinetics, whereas equilibrium data fitted both Langmuir and Freundlich isotherms depending on sludge pretreatment. The significance of this study extends beyond the reported removal efficiencies because it demonstrates that feedstock heterogeneity does not inherently preclude the production of high-performing carbon materials. More importantly, the contrasting surface areas obtained from different sludge conditions indicate that pretreatment itself can become a critical determinant of material performance. Thus, the sustainability advantage of sludge-derived activated carbon depends on whether the additional processing required to control feedstock variability is justified by the resulting functional and environmental benefits. These findings demonstrate that, despite their heterogeneous composition, municipal wastes can produce activated carbons with adsorption performances comparable to those derived from conventional agricultural biomass when appropriate activation procedures are employed.

Beyond pollutant adsorption, biomass-derived activated carbons are increasingly being engineered for multifunctional environmental applications that integrate waste treatment with resource recovery. Chopade and Devatha (2024)^8^ demonstrated that modified areca husk fibre and coconut shell biochars significantly improved dairy sludge conditioning by reducing capillary suction time by 51.6%, decreasing sludge moisture content by 18%, and improving sludge settleability. More importantly, MgO-impregnated biochars recovered 96.6% and 100% of phosphate from dairy sludge using areca husk fibre and coconut shell biochars, respectively. These findings illustrate an important shift in activated carbon research from conventional adsorption toward multifunctional process materials capable of simultaneously improving sludge management, recovering valuable nutrients, and enhancing environmental sustainability. Such multifunctionality is particularly relevant to the sustainability assessment of biomass-derived carbons because the environmental value of a precursor cannot be judged solely by the performance of the final material in a single adsorption experiment. Materials capable of combining pollutant removal, resource recovery, and waste conditioning may provide greater system-level benefits than materials optimized for adsorption capacity alone. Such integrated resource recovery strategies represent an important advancement toward circular economy implementation and broaden the industrial relevance of biomass-derived activated carbons.

These studies demonstrate that precursor selection remains one of the most influential variables governing activated carbon performance. Agricultural residues generally produce carbons with high surface area, favorable pore structures, and excellent mechanical stability owing to their lignocellulosic composition. Animal-derived biomass contributes unique mineral-assisted adsorption mechanisms that expand the range of removable contaminants, whereas industrial and municipal wastes maximize resource recovery and waste valorization despite requiring more rigorous process optimization. However, these categories should not be interpreted as a simple hierarchy of precursor quality. Their relative advantages emerge only when material performance is evaluated against the requirements of a specific application and the burdens associated with production. Therefore, selecting an appropriate biomass should involve a holistic evaluation encompassing precursor chemistry, activation behavior, adsorption performance, regeneration capability, local availability, economic feasibility, and life-cycle sustainability. A major limitation of the existing literature is that these criteria are frequently assessed independently, making it difficult to determine whether a reported high-performance precursor is also the most sustainable option at process or system scale.

This limitation highlights an important direction for future research. Comparative studies should move beyond demonstrating that individual waste-derived precursors can produce activated carbon and instead determine which precursor-activation combinations provide the best balance among carbon yield, functional performance, regeneration, energy and chemical requirements, and life-cycle environmental impacts. Such integrated comparisons are particularly important for establishing whether the apparent environmental advantage of biomass-derived activated carbon is retained when transportation, pretreatment, activation energy, chemical consumption, and end-of-life management are included. Consequently, precursor selection should be treated not simply as a materials-engineering decision but as an early-stage life-cycle decision that can strongly influence the eventual carbon footprint and sustainability of the activated carbon production pathway.

### 3.3 Production technologies

The physicochemical performance of biomass-derived activated carbon is governed not only by the intrinsic characteristics of the precursor but also by the production technology employed. Although biomass composition establishes the initial carbon framework, the conversion route determines pore architecture, surface chemistry, crystallinity, adsorption kinetics, mechanical stability, and regeneration capability. Consequently, the production process represents a major determinant of activated carbon quality and can outweigh precursor effects when processing conditions substantially alter pore development and surface functionality. Activated carbon production generally comprises three stages—carbonization, activation, and post-treatment (Fig. 2)—but their effects are interconnected rather than independent. Changes introduced during carbonization influence the response of the char to activation, while activation conditions subsequently determine the extent to which the resulting structure can deliver adsorption performance. The overall production pathway for converting biomass into activated carbon is illustrated in Fig. 2. The central technological challenge is therefore not simply to maximize surface area, but to obtain the required adsorption performance without imposing disproportionate penalties in carbon yield, energy demand, chemical consumption, cost, or environmental burden.^9,10^

**Fig. 2:**
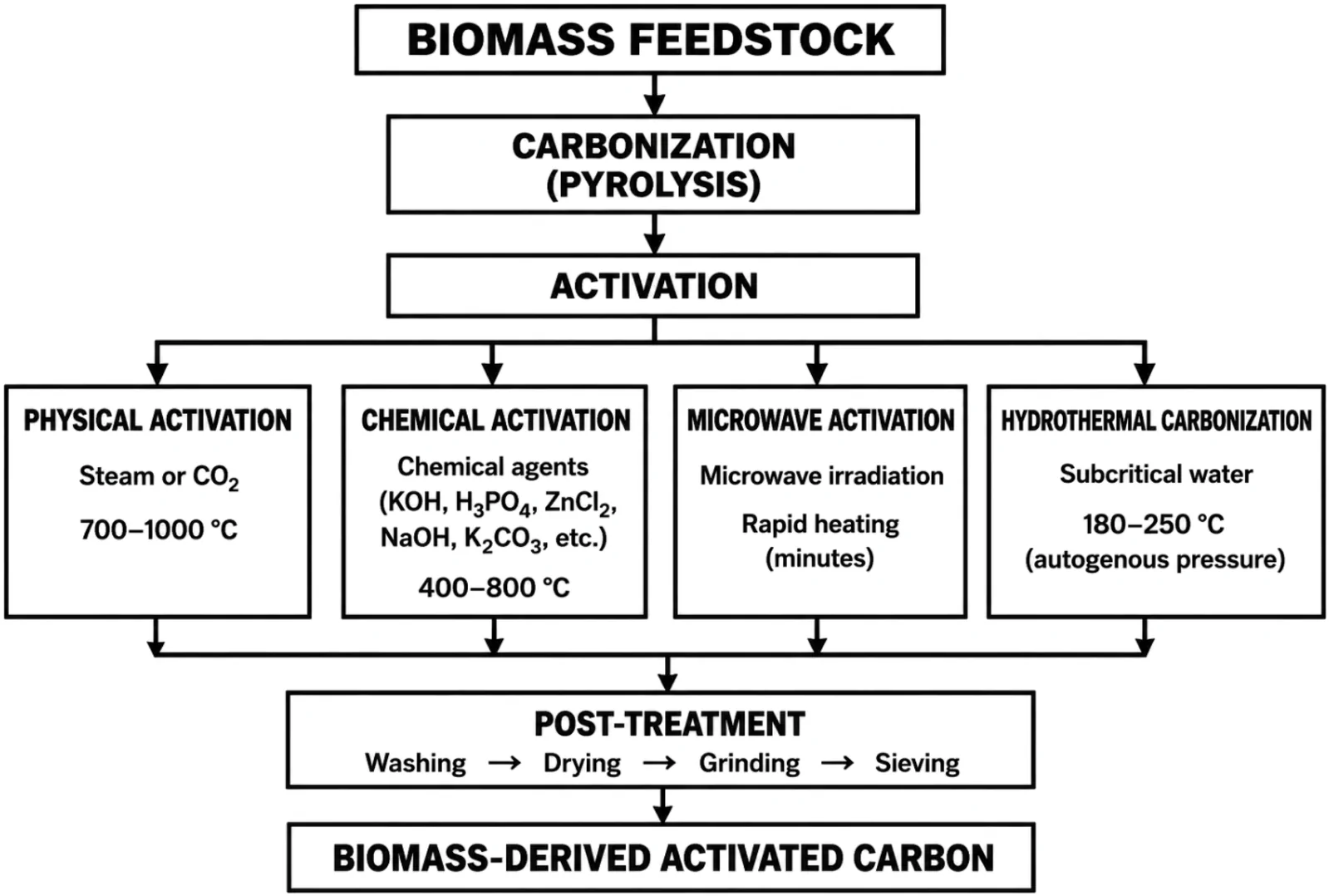
General production pathway for biomass-derived activated carbon.

Carbonization constitutes the initial thermochemical transformation in which biomass is converted into a carbon-rich char through pyrolysis under oxygen-limited or inert conditions, typically between 300 and 700 °C. The decomposition of cellulose, hemicellulose, and lignin removes moisture and volatile constituents while increasing carbon content and aromaticity. However, carbonization alone generally provides an incompletely developed porous structure because tar and other carbonaceous deposits can obstruct emerging pores. It is therefore more appropriately regarded as a structural conditioning stage that determines the quality of the intermediate available for subsequent activation rather than as the principal route for generating high adsorption capacity. Heating rate, residence time, biomass composition, and final temperature consequently become important because they determine whether carbonization produces a char that can be efficiently converted into a highly porous adsorbent.^34,35^

Experimental evidence from Gale *et al.* (2021)^10^ illustrates why carbonization should not be evaluated solely according to conventional indicators such as char yield. The authors compared hydrothermal carbonization (HTC) and slow pyrolysis (SP) of corn stover before potassium hydroxide activation and found that the precursor formation route altered the physicochemical characteristics of the resulting activated carbons. Although HTC reduced the O/C ratio after activation and SP produced comparatively stable elemental characteristics, activated carbon prepared directly from raw corn stover achieved approximately 98% vanillin removal and outperformed the HTC- and SP-derived materials. The finding is significant because it demonstrates that an apparently advantageous pretreatment does not necessarily translate into superior adsorption after activation. Thus, the value of carbonization lies in how effectively it prepares the biomass for downstream pore development rather than in the performance of the carbonized intermediate considered in isolation.

Activation exerts the strongest direct control over the development of adsorption functionality because it converts the relatively compact carbonized matrix into a porous adsorbent. The resulting changes in micropore, mesopore, and macropore development, together with modifications in surface functional groups, directly influence surface area, pore-size distribution, adsorption kinetics, and surface reactivity. Modern approaches include physical, chemical, microwave-assisted, and hydrothermal-assisted routes, but their performance cannot be ranked using surface area alone because gains in textural properties may be accompanied by lower carbon yield, greater energy demand, chemical waste, or poor scalability. A comparative summary of these major production technologies, including their operating principles, advantages, limitations, and typical applications, is presented in Table 4.

**Table 4:** Comparison of major production technologies for biomass-derived activated carbon^9,10,35^

Production stage	Process	Typical operating conditions	Main advantages	Main limitations
Carbonization	Pyrolysis under inert atmosphere	300–700 °C	Produces carbon-rich char; removes volatiles	Limited pore development
Physical activation	Steam or CO_2_ activation	700–1000 °C	No chemical reagents; environmentally cleaner	High energy demand; longer processing time
Chemical activation	KOH, H_3_PO_4_, ZnCl_2_, NaOH, K_2_CO_3_	400–800 °C	High surface area; extensive micropore development	Chemical consumption; wastewater generation
Microwave-assisted activation	Microwave irradiation with or without activating agents	Rapid volumetric heating	Short processing time; improved energy efficiency	Scale-up challenges
Hydrothermal carbonization	Subcritical water treatment	180–250 °C; elevated pressure	Suitable for wet biomass; lower drying requirements	Hydrochar usually requires further activation
Post-treatment	Washing, drying, grinding, sieving	Ambient to moderate temperatures	Improves purity and product quality	Water consumption and additional processing

Physical activation offers an important environmental advantage by employing steam or carbon dioxide rather than chemical activating agents. It consequently reduces dependence on chemical reagents and can simplify downstream purification by limiting chemical residues and secondary wastewater generation. However, the environmental advantage is not inherently equivalent to superior sustainability. Higher temperatures, longer activation periods, and comparatively lower carbon yields can increase energy requirements and offset some of the benefits associated with reagent-free processing. Physical activation is therefore attractive where chemical consumption and wastewater management are major constraints, but its competitiveness depends strongly on the availability of efficient thermal energy and process optimization.

Chemical activation presents the opposite trade-off. Activating agents such as potassium hydroxide, phosphoric acid, zinc chloride, sodium hydroxide, and potassium carbonate can promote pore formation at lower temperatures and, in many cases, generate substantially higher surface areas and micropore volumes. However, the exceptional textural performance achievable through chemical activation may conceal substantial environmental burdens associated with reagent production, impregnation, washing, neutralization, corrosion, and wastewater treatment. Consequently, a process that produces a superior adsorbent on a mass-specific basis cannot automatically be regarded as the more sustainable technology. The relevant question is whether the additional adsorption performance justifies the additional material, energy, and environmental inputs required to obtain it.

The experimental results of Thithai *et al.* (2021)^9^ provide particularly strong evidence for this trade-off. Using coffee waste and oil palm empty fruit bunches, the authors evaluated potassium hydroxide, zinc chloride, and phosphoric acid under different impregnation ratios and carbonization temperatures. Although phosphoric acid produced the highest activated-carbon yield at 600 °C, the maximum BET surface areas—3068 m^2^ g^−1^ and 2147 m^2^ g^−1^—were obtained at 800 °C with potassium hydroxide and zinc chloride, respectively. The accompanying reductions in H/C and O/C ratios indicated increased aromatization and structural ordering at higher temperatures. The results expose a fundamental optimization problem: the conditions that preserve product yield are not necessarily those that maximize porosity, while the conditions producing exceptionally high surface areas can increase carbon loss and energy demand. Activated-carbon production should therefore be optimized against a multidimensional performance criterion rather than the maximum BET value alone.^9^

Microwave-assisted activation offers a potentially different route to this optimization problem by generating volumetric rather than predominantly external heating. Rapid internal heating can shorten processing time and potentially reduce energy consumption while promoting pore development. Its principal limitation, however, is no longer simply material performance but technological translation. Microwave penetration, reactor configuration, process control, feedstock heterogeneity, and scale-up remain important barriers to transferring laboratory advantages to industrial systems. Thus, reported improvements under controlled laboratory conditions should not be interpreted as evidence of established industrial superiority until energy performance and reactor efficiency are demonstrated at relevant scales.

Hydrothermal carbonization similarly illustrates why sustainability claims must be evaluated within the complete production pathway. Its operation under subcritical water conditions of approximately 180–250 °C allows wet biomass to be processed without the energy-intensive drying required before conventional pyrolysis. This makes HTC particularly attractive for sewage sludge, food waste, and other moisture-rich resources. However, hydrochar generally exhibits limited porosity and requires subsequent activation to achieve high adsorption performance. Consequently, HTC does not eliminate the activation problem; rather, it shifts the location and nature of the energy and processing requirements. Its principal advantage may therefore lie in expanding the feedstock range that can be processed efficiently, rather than replacing conventional activation altogether.

Recent work by Ben Salah *et al.* (2026)^35^ provides stronger evidence that process integration may offer greater sustainability gains than optimization of individual stages. Their comparison of conventional two-stage pyrolysis followed by chemical activation with a single-stage simultaneous pyrolysis-activation process showed comparable physicochemical characteristics and methylene blue adsorption capacities. Importantly, the integrated route reduced energy consumption by approximately 24%, carrier gas use by 60%, and processing time by nearly 18%, while both routes achieved almost complete methylene blue removal. The significance of these findings extends beyond the reported adsorption performance: they demonstrate that process simplification can reduce resource requirements without necessarily sacrificing functionality. This challenges the conventional emphasis on maximizing textural properties and supports a shift toward process intensification as a route to improving the practical sustainability of biomass-derived activated carbon.^35^

Post-treatment represents another point at which apparent laboratory performance can diverge from overall process sustainability. Washing, neutralization, drying, grinding, and particle-size classification determine the purity and usability of the final material, but each operation introduces additional resource requirements. Extensive washing can reduce residual activating chemicals but increases water consumption and generates wastewater, whereas insufficient washing may leave residues that compromise product quality or introduce secondary contamination. Similarly, reducing particle size may improve mass-transfer kinetics while weakening mechanical integrity and complicating recovery. These competing effects indicate that post-treatment should be evaluated according to its contribution to the complete life-cycle performance of the adsorbent rather than as a technically necessary but environmentally neutral finishing step.

The evidence indicates that no production technology provides simultaneous maximization of porosity, carbon yield, energy efficiency, process simplicity, and environmental performance. Chemical activation remains capable of producing exceptionally high surface areas, but its reagent and washing requirements can undermine its sustainability advantage. Physical activation reduces chemical inputs but can impose greater thermal demands, while microwave and hydrothermal approaches offer opportunities for process intensification but remain constrained by scale-up and integration challenges. The single-stage approach reported by Ben Salah *et al.* (2026)^35^ further indicates that reducing the number of processing steps may be as important as improving the properties of the final carbon. The overall trajectory of the field is therefore shifting from performance maximization toward systems-level optimization, in which adsorption capacity must be considered alongside carbon yield, regeneration, energy consumption, chemical requirements, scalability, environmental impact, and life-cycle sustainability. Future research should consequently employ standardized multi-criteria assessments rather than relying predominantly on BET surface area or single-contaminant removal efficiency as indicators of technological superiority. Such an approach is essential for determining whether laboratory-scale production innovations can deliver genuine environmental and economic advantages when translated to industrial-scale biomass-derived activated carbon manufacturing.

## 4 Carbon footprint of biomass-derived activated carbon

The increasing demand for sustainable carbon materials has intensified interest in biomass-derived activated carbon as an environmentally preferable alternative to conventional fossil-based activated carbon. Although biomass is generally regarded as a renewable and low-carbon feedstock, the production of activated carbon remains an energy- and resource-intensive process involving multiple stages that contribute to greenhouse gas (GHG) emissions. Consequently, the carbon footprint of biomass-derived activated carbon cannot be evaluated solely on the basis of renewable feedstock utilization but must instead consider emissions generated throughout the entire production chain. These emissions arise from biomass acquisition, transportation, thermal processing, chemical consumption, utility requirements, product finishing, and distribution, all of which collectively determine the overall climate impact of the material.

Unlike conventional environmental assessments that focus primarily on direct process emissions, carbon footprint analysis considers both direct and indirect sources of greenhouse gas emissions associated with material production. Direct emissions originate from on-site operations such as biomass drying, carbonization, and activation, whereas indirect emissions are associated with electricity generation, chemical manufacturing, transportation, equipment operation, and supporting infrastructure. The relative contribution of each emission source varies considerably depending on biomass type, production technology, energy source, plant scale, and geographical location. Therefore, identifying emission hotspots within the manufacturing process is essential for developing low-carbon production strategies and improving the environmental performance of biomass-derived activated carbon.

### 4.1 System boundaries for carbon footprint assessment

Accurate quantification of the carbon footprint of biomass-derived activated carbon depends fundamentally on the definition of appropriate system boundaries, which determine the life-cycle stages included in the assessment and directly influence the magnitude of reported greenhouse gas (GHG) emissions. Although carbon footprint analysis has become an indispensable tool for evaluating the environmental sustainability of materials and manufacturing processes, inconsistencies in boundary selection remain one of the principal reasons for the wide variability observed among published studies.^36,37^ Consequently, differences in reported emissions frequently reflect methodological choices rather than genuine improvements in production technology. This limitation has become increasingly apparent in life-cycle assessments of biomass-derived materials, where identical production systems may exhibit substantially different carbon footprints depending on whether upstream raw material acquisition, downstream product use, regeneration, or end-of-life management are included within the assessment framework. Therefore, selecting transparent and standardized system boundaries is essential for ensuring methodological consistency, facilitating meaningful comparisons among production systems, and supporting evidence-based environmental decision-making. Three principal system boundary approaches are commonly adopted for biomass-derived activated carbon, namely cradle-to-gate, cradle-to-grave, and cradle-to-cradle assessments, each providing different perspectives on environmental performance (Table 5).^38,39^

**Table 5:** Common system boundaries used for carbon footprint assessment of biomass-derived activated carbon^36–43^

System boundary	Included life-cycle stages	Typical application	Implication for carbon-negative assessment
Cradle-to-gate	Biomass collection to activated carbon production	Manufacturing/process comparison	Does not capture use, regeneration or end-of-life carbon fate
Cradle-to-grave	Production, transport, use, regeneration and disposal	Full product assessment	More appropriate for evaluating whole-life carbon balance
Cradle-to-cradle	Production, use, regeneration, recycling/material recovery	Circular systems	Potentially suitable for assessing carbon retention and repeated use

Among these approaches, the cradle-to-gate boundary remains the most frequently adopted because it evaluates emissions generated from biomass acquisition through activated carbon production up to the factory gate. This boundary generally encompasses biomass collection, transportation, preprocessing, carbonization, activation, post-treatment, and packaging while excluding emissions associated with product distribution, operational use, regeneration, and disposal. Owing to its relatively straightforward implementation and lower data requirements, cradle-to-gate assessment is particularly valuable for comparing manufacturing technologies and identifying production-stage emission hotspots. Nevertheless, its restricted scope inevitably underestimates the total environmental burden because downstream processes may contribute substantially to life-cycle greenhouse gas emissions, particularly for products designed for repeated regeneration and long-term service.^40–42^

The limitations associated with narrow assessment boundaries have been increasingly demonstrated through recent life-cycle investigations. Carrejo *et al.* (2026),^36^ in evaluating carbon capture integration within kraft pulp and paper production, reported that system boundary selection exerted a profound influence on the perceived environmental performance of the technology. When evaluated using a gate-to-gate perspective that considered only emissions associated with the carbon capture facility, carbon removal efficiencies reached approximately 92%. However, extending the assessment to cradle-to-grave conditions, incorporating upstream forestry operations, mill-wide emissions, and downstream product decomposition, reduced removal efficiencies to between 17% and 46%, with further reductions observed when dynamic biogenic carbon accounting was incorporated. These findings clearly demonstrate that reported environmental performance can vary dramatically without any change in process technology, solely as a consequence of methodological choices. Although this study focused on the pulp and paper industry, its implications are directly applicable to biomass-derived activated carbon, where inconsistencies in boundary definition continue to hinder objective comparison among feedstocks, activation technologies, and manufacturing routes.

While cradle-to-gate assessment remains valuable for process optimization, it provides only a partial representation of environmental sustainability because activated carbon commonly undergoes multiple regeneration cycles before disposal. Consequently, cradle-to-grave assessment has gained increasing attention as a more comprehensive framework capable of capturing emissions associated with transportation, operational use, regeneration, recycling, and end-of-life treatment. This broader perspective is particularly relevant for activated carbon because regeneration substantially prolongs service life and reduces the demand for virgin adsorbent production. However, cradle-to-grave analyses are inherently associated with greater uncertainty because regeneration efficiency, transportation distances, operational lifespan, and disposal practices vary considerably among applications and geographical regions. Accordingly, although cradle-to-grave assessment offers a more realistic representation of environmental performance, its reliability depends heavily on the availability of robust inventory data and well-defined modelling assumptions.

The growing transition toward circular manufacturing has further expanded the application of cradle-to-cradle assessment, which incorporates resource recovery, recycling, material recirculation, and product regeneration within the system boundary. Rather than considering spent activated carbon as waste, cradle-to-cradle analysis recognizes opportunities for thermal reactivation, secondary utilization, and energy recovery that reduce dependence on virgin biomass while minimizing cumulative greenhouse gas emissions. This perspective is particularly compatible with biomass-derived activated carbon because most precursor materials originate from agricultural, forestry, or industrial residues that are themselves products of circular resource utilization. Consequently, cradle-to-cradle assessment extends beyond conventional environmental accounting by simultaneously evaluating waste valorization, carbon sequestration, material recovery, and long-term resource efficiency.

Despite the conceptual advantages of broader system boundaries, their implementation remains challenging because increasing boundary complexity requires larger datasets, additional modelling assumptions, and greater computational effort. Cambazoğlu *et al.* (2025),^37^ for example, quantified the cradle-to-gate carbon footprint of melamine-faced particleboard and demonstrated that Scope 3 emissions accounted for approximately 67% of total greenhouse gas emissions, substantially exceeding emissions associated with direct manufacturing operations. Transportation of raw materials, electricity consumption, and adhesive production were identified as the dominant contributors, emphasizing that upstream supply-chain activities frequently outweigh direct process emissions. Similar trends are expected for biomass-derived activated carbon, where biomass transportation, production of activating agents such as potassium hydroxide and phosphoric acid, and electricity consumed during thermal processing may collectively contribute a significant proportion of total greenhouse gas emissions. These findings highlight the importance of extending carbon footprint analyses beyond reactor-scale operations to include upstream resource acquisition and supporting industrial processes.

Methodological inconsistencies extend beyond system boundary selection to encompass functional units, allocation procedures, emission factors, geographical assumptions, and inventory quality. Kennelly *et al.* (2019)^38^ demonstrated that incomplete process-based carbon accounting models may underestimate supply-chain emissions by as much as 60% owing to truncation errors arising from inadequate system boundaries. Similarly, Pan *et al.* (2018)^39^ reported that inconsistencies in temporal, spatial, functional, and methodological boundaries were responsible for significant discrepancies among building life-cycle carbon assessments, limiting meaningful benchmarking between studies. Comparable observations were reported by Liu *et al.* (2015),^40^ who demonstrated that regional carbon emission inventories varied substantially depending on whether production-based or consumption-based accounting approaches were adopted. Collectively, these investigations indicate that discrepancies among published carbon footprint studies frequently arise from methodological inconsistency rather than differences in technological performance, reinforcing the need for harmonized life-cycle assessment protocols.

The importance of standardized assessment methodologies has also been emphasized in agricultural and environmental carbon accounting. Adewale *et al.* (2018)^41^ argued that inconsistent system boundaries, generalized emission factors, and inadequate consideration of soil carbon dynamics frequently produce fundamentally different estimates of agricultural carbon footprints, thereby reducing comparability among studies and potentially leading to misguided policy decisions. Likewise, Pasek *et al.* (2023)^42^ demonstrated that methodological variations involving system boundaries, functional units, geographical averaging, and data availability substantially influence carbon footprint estimates within the information and communications technology sector. Although these investigations were conducted outside biomass-derived activated carbon production, they collectively reinforce a common conclusion: carbon footprint outcomes are highly sensitive to methodological design, and robust comparisons require transparent, standardized assessment frameworks.

More recently, broader sustainability frameworks have further expanded conventional carbon accounting by integrating planetary boundary concepts into environmental assessment. Yue *et al.* (2023)^43^ demonstrated that combining ecological footprint analysis with planetary boundary theory provides a more comprehensive understanding of resource consumption, carbon emissions, and ecological carrying capacity than conventional carbon accounting alone. Although such integrated approaches have yet to be widely adopted in biomass-derived activated carbon research, they offer considerable potential for future sustainability assessments by simultaneously evaluating greenhouse gas emissions, resource depletion, land use, and ecosystem resilience within a unified analytical framework.

Current evidence demonstrates that the definition of system boundaries represents one of the most influential methodological decisions in carbon footprint assessment. Narrow boundaries facilitate process optimization but frequently underestimate environmental impacts, whereas broader life-cycle approaches provide more comprehensive sustainability evaluations at the expense of increased methodological complexity and data requirements. Consequently, future investigations should move beyond isolated carbon footprint calculations toward internationally harmonized assessment protocols incorporating standardized system boundaries, consistent functional units, transparent inventory reporting, and integrated life-cycle methodologies. Such harmonization will substantially improve the reliability, comparability, and practical relevance of carbon footprint assessments for biomass-derived activated carbon while providing a more robust scientific basis for technology development, environmental policy formulation, and industrial implementation.

### 4.2 Major carbon emission sources throughout the production chain

The carbon footprint of biomass-derived activated carbon arises from an interconnected production chain extending from biomass acquisition to product distribution. Although biomass provides a renewable carbon source, its conversion into activated carbon requires energy, transportation, chemicals, and water, meaning that renewability of the precursor does not inherently translate into a low-carbon product. A meaningful carbon assessment must therefore account for emissions across the entire production chain rather than focusing exclusively on the conversion reactor.^34^

Biomass acquisition is an important upstream determinant of emissions because its environmental burden depends on feedstock origin, collection requirements, and logistics. Agricultural residues can offer an advantage because they are generated as by-products rather than requiring dedicated cultivation, whereas purpose-grown biomass may introduce additional cultivation, fertilizer, irrigation, and harvesting burdens. However, this advantage can be weakened when residues have low bulk density, high moisture content, or require long-distance transport. Thus, feedstock selection should be evaluated together with supply-chain configuration rather than treated as an inherently sustainable attribute.^44^ From a carbon-negative perspective, the benefit of using a residue therefore depends not only on its renewable or waste status but also on the emissions avoided by diverting it from its alternative fate and the additional emissions generated during its collection and conversion.

Transportation can further erode the environmental advantage of biomass when feedstocks are dispersed or inefficiently transported. Emissions depend on distance, moisture content, bulk density, transport mode, vehicle efficiency, and infrastructure. This makes the geographical relationship between biomass availability and processing facilities a potentially important carbon-footprint determinant. Decentralized processing and use of locally available residues may therefore provide greater emission reductions than simply substituting fossil-derived precursors with biomass.^45^ Accordingly, minimizing transport-related emissions can strengthen carbon-negative potential, whereas long-distance transport may reduce or even eliminate the net carbon benefit of biomass conversion.

The broader significance of supply-chain emissions is illustrated by Davis *et al.* (2011),^44^ who showed that substantial proportions of global fossil-fuel-related CO_2_ emissions and embodied emissions are associated with internationally traded fuels, goods, and services. Although these estimates are not specific to activated carbon, they demonstrate why restricting carbon accounting to the production facility can systematically underestimate upstream emissions. Activated-carbon LCAs should consequently include feedstock transport and other embodied supply-chain contributions rather than treating them as peripheral processes.^44^ Failure to account for these upstream burdens could result in an apparently favourable carbon balance that does not represent the full life-cycle performance required to support a carbon-negative claim.

Drying represents another potentially significant burden, particularly for high-moisture feedstocks such as sewage sludge and food-processing residues. Its contribution is governed largely by the initial moisture content and energy source. Consequently, the environmental benefit of selecting a waste biomass can be substantially reduced when large amounts of energy are required simply to remove water before carbonization. Waste-heat recovery, solar drying, and improved dewatering therefore represent important opportunities for reducing the upstream energy requirement.^34^ Lowering the energy demand associated with drying can directly improve the net carbon balance, particularly where drying energy is supplied from fossil-intensive sources.

The most consequential production-stage trade-off occurs during carbonization and activation. Both processes require substantial thermal energy, while chemical activation additionally introduces burdens associated with the manufacture, transport, use, recovery, and disposal of activating agents. Importantly, the pursuit of exceptionally high surface area may increase rather than decrease environmental impacts because greater activation intensity can require higher energy and chemical inputs. Thus, maximizing adsorption performance without simultaneously accounting for energy and chemical demand may produce materials that are functionally superior but environmentally less favorable. Because carbonization and activation determine both the energy demand and the amount and properties of carbon retained in the final product, their operating conditions are central to determining whether the resulting activated carbon can deliver a net climate benefit.

The importance of interconnected emission pathways is supported by Jiang *et al.* (2021),^45^ whose carbon-flow analysis showed that emissions can become concentrated within interconnected upstream sectors rather than being attributable to individual production units. Applied to biomass-derived activated carbon, this perspective indicates that electricity generation, thermal processing, chemical manufacture, and biomass logistics should be evaluated as coupled contributors to the final carbon footprint. Carbon-reduction strategies should therefore target dominant upstream emission nodes rather than optimizing individual processing steps in isolation.^45^ Such integrated emission reduction is particularly important for carbon-negative potential because reductions achieved at one stage may be offset by burdens generated elsewhere in the production chain.

Post-treatment also contributes to the overall environmental burden through washing, neutralization, drying, grinding, and wastewater management. Although these operations may be smaller contributors than carbonization and activation, their significance increases when chemical activation requires extensive washing or when wastewater treatment is energy- and chemical-intensive. Consequently, post-treatment cannot be excluded from a complete carbon-footprint assessment simply because it occurs after the principal thermal conversion step.^34^ Reducing water, chemical, and energy requirements during post-treatment can therefore improve the overall carbon balance and increase the likelihood that the product's carbon-storage or substitution benefits exceed its attributable life-cycle emissions.

Downstream packaging, storage, and distribution represent additional embodied emissions that become increasingly relevant when activated carbon is transported over long distances. Their contribution is particularly important for internationally traded products, where the environmental advantage achieved during biomass conversion may be partly offset by distribution requirements. Regional production, efficient logistics, and reduced packaging can therefore complement process-level emission reduction.^34^ For a carbon-negative system, these downstream emissions must also be included when determining whether the cumulative climate benefits of the activated carbon remain greater than its total life-cycle burdens.

Evidence from other bioresource production systems reinforces the need for integrated mitigation. Yang *et al.* (2023)^34^ identified multiple interacting emission hotspots across pork production and reported that combined interventions could reduce emissions by 6.5–50%, depending on the production stage. Although the system differs from activated-carbon production, the finding supports a broader LCA principle: substantial reductions are more likely when several interconnected hotspots are addressed simultaneously rather than when a single process is optimized. This principle is directly relevant to carbon-negative potential because the achievement of a net-negative balance depends on the cumulative effect of emission reductions across the entire production chain rather than on the performance of any individual stage.

The carbon footprint is determined by the interaction of feedstock logistics, process energy, activation intensity, chemical consumption and downstream operations, highlighting the need for whole-chain optimization rather than isolated process improvements.

### 4.3 Carbon accounting

Standardized under the ISO 14040 and ISO 14044 series, LCA assesses the environmental performance of products and processes by quantifying resource consumption, energy requirements, emissions, and environmental impacts across defined life-cycle stages. For biomass-derived activated carbon, LCA is particularly important because environmental performance depends not only on the conversion process but also on biomass sourcing, transportation, preprocessing, activation chemistry, energy supply, product use, regeneration, and end-of-life management.^14–16^ However, comparisons among published studies remain challenging because researchers frequently adopt different system boundaries, functional units, allocation procedures, emission factors, geographical assumptions, and inventory datasets. These methodological differences can substantially influence reported carbon footprints and may produce apparently conflicting conclusions even when similar technologies or feedstocks are assessed.

The selection of system boundaries represents one of the most influential decisions in carbon footprint assessment. Cradle-to-gate studies typically include biomass acquisition, transportation, preprocessing, carbonization, activation, and product manufacturing, but terminate the assessment before the activated carbon enters its intended application. Such boundaries are useful for comparing production technologies but may omit important downstream processes, including adsorption, regeneration, reuse, and final disposal. Cradle-to-grave assessments extend the boundary to product use and end-of-life, thereby providing a more complete representation of the environmental burden associated with the activated carbon. Cradle-to-cradle approaches may further incorporate recycling, regeneration, material recovery, and other circular pathways. For assessments specifically intended to support claims of carbon-negative performance, broader system boundaries are generally more informative because a negative production-stage footprint does not necessarily imply a negative whole-life GHG balance.^17^

The counterfactual fate of the biomass feedstock is another critical consideration. Biomass residues cannot automatically be treated as atmospheric carbon removal simply because they are classified as wastes. The climate benefit of converting a residue into activated carbon depends partly on what would have occurred in the absence of the proposed conversion pathway—for example, whether the biomass would have decomposed, been openly burned, used for energy recovery, incorporated into soil, or diverted to another material application. Consequently, the avoided emissions or carbon benefits associated with biomass valorization should be based on a clearly defined and credible counterfactual rather than assumed solely from the renewable origin of the feedstock.^18,20^

An important consideration in determining the carbon-negative potential of biomass-derived activated carbon is the counterfactual fate of the biomass feedstock. The classification of a material as a “waste” or “residue” does not, by itself, establish an atmospheric carbon benefit from its conversion into activated carbon. The appropriate baseline depends on what would have occurred in the absence of the proposed conversion pathway. For example, a residue that would otherwise decompose may have a different greenhouse-gas profile from one that would be openly burned, used for energy recovery, or diverted to another material application. Similarly, where a residue already has an established productive use, its allocation to activated-carbon production may generate opportunity or substitution effects that should be incorporated into the assessment. Therefore, the climate benefit attributable to biomass-waste valorization should be evaluated relative to a clearly defined counterfactual scenario rather than assuming that all carbon contained in the residue represents additional atmospheric carbon removal.^17–20^ This distinction is particularly important for carbon-negative claims because the net climate benefit depends on the difference between emissions under the activated-carbon pathway and those associated with the biomass's plausible alternative fate.

Despite the conceptual advantages of broader system boundaries, their implementation remains challenging because increasing boundary complexity requires larger datasets, additional modelling assumptions, and greater computational effort. Cambazoğlu *et al.* (2025)^37^, for example, quantified the cradle-to-gate carbon footprint of melamine-faced particleboard and demonstrated that Scope 3 emissions accounted for approximately 67% of total greenhouse gas emissions, substantially exceeding emissions associated with direct manufacturing operations. Transportation of raw materials, electricity consumption, and adhesive production were identified as the dominant contributors, emphasizing that upstream supply-chain activities frequently outweigh direct process emissions. Similar trends are expected for biomass-derived activated carbon, where biomass transportation, production of activating agents such as potassium hydroxide and phosphoric acid, and electricity consumed during thermal processing may collectively contribute a significant proportion of total greenhouse gas emissions. These findings highlight the importance of extending carbon footprint analyses beyond reactor-scale operations to include upstream resource acquisition and supporting industrial processes.

Methodological inconsistencies extend beyond system boundary selection to encompass functional units, allocation procedures, emission factors, geographical assumptions, and inventory quality. Kennelly *et al.* (2019)^38^ demonstrated that incomplete process-based carbon accounting models may underestimate supply-chain emissions by as much as 60% owing to truncation errors arising from inadequate system boundaries. Similarly, Pan *et al.* (2018)^39^ reported that inconsistencies in temporal, spatial, functional, and methodological boundaries were responsible for significant discrepancies among building life-cycle carbon assessments, limiting meaningful benchmarking between studies. Comparable observations were reported by Liu *et al.* (2015),^40^ who demonstrated that regional carbon emission inventories varied substantially depending on whether production-based or consumption-based accounting approaches were adopted. These investigations indicate that discrepancies among published carbon footprint studies frequently arise from methodological inconsistency rather than differences in technological performance, reinforcing the need for harmonized life-cycle assessment protocols.

The importance of standardized assessment methodologies has also been emphasized in agricultural and environmental carbon accounting. Adewale *et al.* (2018)^41^ argued that inconsistent system boundaries, generalized emission factors, and inadequate consideration of soil carbon dynamics frequently produce fundamentally different estimates of agricultural carbon footprints, thereby reducing comparability among studies and potentially leading to misguided policy decisions. Likewise, Pasek *et al.* (2023)^42^ demonstrated that methodological variations involving system boundaries, functional units, geographical averaging, and data availability substantially influence carbon footprint estimates within the information and communications technology sector. Although these investigations were conducted outside biomass-derived activated carbon production, they collectively reinforce a common conclusion: carbon footprint outcomes are highly sensitive to methodological design, and robust comparisons require transparent, standardized assessment frameworks. Table 6 summarizes the key methodological factors that influence life-cycle carbon assessment of biomass-derived activated carbon.

**Table 6:** Methodological factors influencing life-cycle carbon assessment of biomass-derived activated carbon

LCA factor	Evidence in the reviewed literature	Relevance to biomass-derived activated carbon	Implication for carbon-footprint interpretation
System boundary	Different boundary configurations can substantially alter calculated carbon performance^36,38–40^	Should clearly define whether biomass acquisition, transport, production, use, regeneration and EOL are included	Results from different boundaries should not be directly compared without adjustment
Functional unit	Carbon assessments use different functional bases depending on the product/system^37–41^	Activated carbon should ideally be compared using a functional unit reflecting equivalent service, not simply mass of product	Different functional units can produce apparently conflicting results
Biomass/residue treatment	Agricultural and biomass systems require explicit treatment of feedstock origin and associated burdens^41^	Residues should be assessed according to their actual counterfactual fate and collection burden	“Waste biomass” should not automatically be assigned zero environmental burden
Allocation/substitution	Carbon accounting outcomes can depend on allocation and system-expansion assumptions^38,40,41^	Co-products and avoided products should be treated consistently	Carbon benefits may change substantially depending on allocation/substitution
Energy/electricity	Upstream energy and supply-chain emissions can contribute significantly to overall carbon footprints^44,45^	Electricity source and thermal-energy supply should be explicitly reported	Renewable electricity or process heat can substantially alter the carbon footprint
Biogenic carbon	Carbon accounting frameworks differ in how carbon flows and storage are represented^36,40,41^	Biomass carbon uptake, conversion, storage and release should be tracked consistently	Biogenic carbon uptake alone does not establish carbon negativity
Regeneration and EOL	Downstream stages can materially influence life-cycle performance^40–42^	Activated carbon assessments should include regeneration frequency, energy demand and final disposal/reuse where relevant	Cradle-to-gate results may overstate environmental benefits
Supply-chain emissions	Interconnected upstream sectors can be important sources of emissions^44,45^	Feedstock transport, chemical manufacture and electricity generation should be included	Local sourcing and low-carbon inputs may be important mitigation strategies
Uncertainty	LCA outcomes are sensitive to modelling assumptions and system configuration^36–42^	Sensitivity/uncertainty analysis should accompany comparative claims	Apparent technological advantages should be distinguished from modelling effects

More recently, broader sustainability frameworks have further expanded conventional carbon accounting by integrating planetary boundary concepts into environmental assessment. Yue *et al.* (2023)^43^ demonstrated that combining ecological footprint analysis with planetary boundary theory provides a more comprehensive understanding of resource consumption, carbon emissions, and ecological carrying capacity than conventional carbon accounting alone. Although such integrated approaches have yet to be widely adopted in biomass-derived activated carbon research, they offer considerable potential for future sustainability assessments by simultaneously evaluating greenhouse gas emissions, resource depletion, land use, and ecosystem resilience within a unified analytical framework.

Biogenic carbon accounting requires particular attention when interpreting the carbon balance of biomass-derived activated carbon. Carbon incorporated into the activated-carbon matrix should not automatically be equated with permanent atmospheric CO_2_ removal. The relevant assessment must distinguish between carbon temporarily retained during the product life cycle and carbon that remains durably stored over an appropriately defined time horizon. Carbon stability, potential degradation or oxidation, combustion, regeneration, and end-of-life treatment can determine whether carbon initially retained in the activated carbon remains sequestered or is ultimately returned to the atmosphere.

Regeneration introduces an additional trade-off. Reuse and regeneration can reduce the demand for new activated carbon by extending adsorbent service life, potentially lowering cumulative production-related emissions. However, regeneration may require substantial heat, electricity, water, or chemical inputs and may itself generate additional emissions. Its contribution to climate performance should therefore be evaluated over the complete service life of the adsorbent rather than assumed to be beneficial solely because the material is reusable.

Similarly, end-of-life treatment should be incorporated when sufficient data are available. Reuse, regeneration, material recovery, combustion, landfill disposal, or other end-of-life pathways can produce substantially different carbon outcomes. In particular, combustion or degradation of carbon-rich spent adsorbents may return previously retained carbon to the atmosphere, whereas pathways that maintain carbon within stable solid forms may provide greater long-term retention. Accordingly, carbon-negative claims should be based on the carbon fate of the activated carbon after use rather than on carbon content measured immediately after production.

Allocation and substitution assumptions also require careful scrutiny. When activated-carbon production generates coproducts such as bio-oil, syngas, heat, or other valuable streams, the choice of mass, energy, economic, or system-expansion allocation can alter the emissions attributed to the activated-carbon product. Similarly, avoided-emission credits from substituting conventional activated carbon should be based on a realistic and clearly specified reference product, including differences in performance, service lifetime, regeneration requirements, and end-of-life treatment. Such credits should therefore not be interpreted as direct atmospheric carbon removal.

Recent empirical evidence further demonstrates that methodological choices can influence calculated emissions as much as technological improvements. Wang *et al.* (2025),^46^ through a large-scale evaluation of 49 wastewater treatment plants, compared different carbon accounting methodologies and reported that calculated greenhouse gas emissions differed by 19.7% between the Standard for Carbon Emission Reduction Assessment of Urban Wastewater Treatment Plants (GS 2023) and the IPCC (2019) methodology. More importantly, indirect emissions accounted for 56.8% of total emissions, with electricity consumption contributing 43.3% and chemical utilization 13.5%, emphasizing that upstream energy production and material inputs frequently dominate overall carbon footprints. The study further revealed substantial variability among emission factors, including a 236.9% difference in carbon-source emission coefficients, demonstrating that inaccurate emission factors may introduce uncertainties comparable to those arising from technological differences. Although conducted within wastewater treatment systems, these findings are directly relevant to biomass-derived activated carbon production, where electricity generation and activating chemical manufacture likewise constitute major indirect emission sources.

Beyond emission factors, energy intensity has become an increasingly important performance indicator because it links process efficiency directly with greenhouse gas emissions. Energy intensity expresses the quantity of energy required to produce a unit mass of activated carbon and therefore provides an indirect measure of process sustainability. Carbonization and activation consistently exhibit the highest energy demand owing to prolonged exposure to elevated temperatures, making them the principal contributors to operational carbon emissions in most production systems. Nevertheless, interpreting energy intensity in isolation may be misleading because lower energy consumption does not necessarily translate into lower life-cycle emissions if the energy source possesses a high carbon intensity. Consequently, reducing the environmental footprint of activated carbon production requires simultaneous consideration of both energy efficiency and the carbon intensity of the energy supply. Strategies such as waste heat recovery, process intensification, renewable electricity integration, microwave-assisted heating, and optimized activation conditions therefore offer opportunities to reduce greenhouse gas emissions while maintaining product performance.

Recent developments in carbon accounting have also shifted attention from static emission inventories toward dynamic, real-time monitoring systems capable of providing continuous carbon emission measurements. Ding *et al.* (2025)^47^ highlighted the growing importance of real-time carbon metering in improving accounting accuracy through Continuous Emission Monitoring Systems (CEMS), advanced sensor technologies, and artificial intelligence-assisted data interpretation. Their review demonstrated that emerging technologies, including non-dispersive infrared (NDIR) sensors, nanotechnology-enabled sensing platforms, and machine learning algorithms, substantially improve the sensitivity, reliability, and temporal resolution of carbon measurements compared with conventional inventory-based approaches. However, widespread implementation remains constrained by high installation costs, technological complexity, and regional variations in emission reporting standards. These observations suggest that future carbon accounting for biomass-derived activated carbon should gradually transition from static life-cycle inventories toward dynamic monitoring systems capable of providing continuous process-level emission data, thereby enhancing both the precision and transparency of environmental assessments.

Temporal aspects are particularly important when carbon storage is used to support a carbon-negative claim. Carbon retained in activated carbon for a short period should not necessarily be assigned the same climate benefit as carbon expected to remain stored for decades or longer. The appropriate accounting horizon should therefore be stated explicitly, and claims of durable carbon storage should be supported by evidence concerning carbon stability and likely release pathways. Where such information is unavailable, the carbon-storage contribution should be treated conservatively rather than assumed to represent permanent removal.

Despite considerable methodological advances, several challenges continue to limit the reliability and comparability of carbon accounting studies. Variability in system boundaries, functional units, emission factors, allocation procedures, data quality, and regional electricity mixes frequently produces inconsistent carbon footprint estimates, even for identical production technologies. Furthermore, most current assessments emphasize total greenhouse gas emissions while giving comparatively little attention to temporal carbon dynamics, biogenic carbon storage, uncertainty propagation, and indirect supply-chain emissions. These limitations are particularly consequential when negative carbon balances are reported because uncertainty in carbon storage, counterfactual scenarios, emission factors, energy inputs, allocation, and end-of-life pathways may determine whether an apparently negative result remains negative under alternative assumptions. Consequently, negative values should be interpreted according to the completeness and robustness of the underlying inventory and sensitivity analysis rather than as definitive evidence of atmospheric carbon removal.

The carbon-accounting evidence supports the potential for biomass-derived activated carbon to achieve lower life-cycle GHG emissions and, under favourable and sufficiently comprehensive conditions, a net negative GHG balance. However, carbon negativity should not be inferred solely from renewable feedstock origin, waste valorization, carbon content, or comparison with fossil-derived activated carbon. A defensible carbon-negative claim requires that durable carbon storage or removal, together with any credible substitution benefits, exceeds all attributable life-cycle emissions under clearly defined system boundaries and assumptions. Where key elements such as counterfactual biomass fate, carbon permanence, regeneration, end-of-life emissions, indirect supply-chain effects, or uncertainty are inadequately characterized, the evidence should instead be described as indicating carbon-negative potential rather than demonstrated carbon-negative performance.

#### 4.3.1 Framework for assessing carbon-negative potential

Carbon-negative potential should be distinguished from simply achieving a lower carbon footprint through renewable feedstocks or waste valorization. A lower-carbon system generates fewer life-cycle greenhouse-gas (GHG) emissions than a defined comparator, whereas a carbon-neutral system approximately balances attributable emissions and removals within a specified accounting boundary. A system should be considered carbon-negative only when durable carbon storage or removal exceeds the total attributable life-cycle GHG emissions. Therefore, the use of biomass residues or a lower carbon footprint relative to fossil-derived activated carbon should not, by itself, be interpreted as evidence of carbon-negative performance.^14–20^

For the purpose of this review, the net carbon balance can be conceptually expressed as:GHG_net_ = GHG_LC_ − *C*_durable_ − GHG_avoided_where GHG_net_ is the net life-cycle GHG impact, GHG_LC_ represents total attributable life-cycle GHG, emissions, *C*_durable_ represents durable carbon removal or storage, and GHG_avoided_ represents credible avoided emissions associated with substitution or displacement. Under this framework, a negative GHG_net_ indicates a net carbon-negative balance.^17–20^

Under this framework, (GHG_net_ < 0) indicates a net carbon-negative balance. The life-cycle emissions should, where relevant, include biomass collection and transportation, pretreatment, drying, carbonization, activation, chemical inputs, electricity and heat requirements, product use, regeneration, and end-of-life management. This approach is consistent with the broader LCA perspective adopted in this review, in which environmental outcomes are strongly influenced by system boundaries, functional units, allocation procedures, emission factors, geographical assumptions, and inventory quality.^17–20,37–42^

Biogenic carbon accounting is particularly important when assessing carbon-negative potential. Carbon contained in biomass-derived activated carbon should not automatically be regarded as permanent atmospheric carbon removal. The appropriate assessment must consider the counterfactual fate of the biomass, including whether the residue would otherwise have decomposed, been burned, used for energy, or diverted to another material application. Similarly, the stability and permanence of carbon retained in the activated carbon must be considered over the relevant time horizon. Carbon temporarily retained during product use may ultimately be released through combustion, degradation, regeneration, or other end-of-life pathways. Consequently, carbon retention during the service life of the adsorbent should be distinguished from durable carbon storage.

Regeneration further illustrates the importance of whole-life accounting. Although effective regeneration can extend adsorbent lifetime and reduce the need for repeated production of fresh activated carbon, regeneration itself may require additional heat, electricity, water, or chemicals and may generate further emissions. Its contribution to the net carbon balance therefore depends on regeneration frequency, process efficiency, energy source, and the resulting extension of product lifetime. Similarly, end-of-life management can substantially influence the final carbon balance because reuse, regeneration, combustion, landfilling, or other disposal pathways have different implications for carbon retention and GHG emissions.

Allocation and substitution assumptions also require careful consideration. Where activated-carbon production generates coproducts such as heat, bio-oil, or syngas, the allocation procedure can influence the emissions attributed to the activated-carbon product. Likewise, avoided-emission credits associated with replacing conventional activated carbon should be based on a clearly defined and realistic reference product rather than being assumed solely because the biomass-derived product is renewable. These methodological choices can therefore produce substantial differences between apparently similar carbon-footprint estimates, consistent with the methodological variability identified in existing LCA studies.^17–20,37–42^

On this basis, the evidence reviewed here is interpreted according to the strength of the available carbon-accounting evidence. Evidence that biomass-derived activated carbon has a lower carbon footprint than a conventional alternative supports a lower-carbon classification but does not establish carbon negativity. LCA studies that report a negative net balance under clearly defined assumptions provide evidence of potential carbon-negative performance, whereas stronger claims require comprehensive accounting of upstream and downstream emissions together with credible treatment of carbon permanence, counterfactual biomass fate, regeneration, end-of-life pathways, and uncertainty. Thus, the term “carbon-negative potential” is used in this review to describe a conditional environmental opportunity rather than an inherent property of all biomass-derived activated carbon systems.

### 4.4 Environmental hotspots

Although biomass-derived activated carbon is widely recognized as a more sustainable alternative to fossil-derived activated carbon because of its renewable feedstocks and waste valorization potential, its environmental performance is determined less by biomass origin than by the cumulative impacts of the production system. Life-cycle assessment (LCA) studies consistently demonstrate that greenhouse gas emissions, energy consumption, and resource depletion are concentrated within a limited number of energy- and material-intensive processes rather than being uniformly distributed across the production chain.^48^ Consequently, identifying environmental hotspots is fundamental for improving process efficiency, minimizing carbon emissions, and guiding the development of low-impact activated carbon production technologies. However, comparison among published studies remains challenging because environmental hotspot identification is strongly influenced by methodological choices, including system boundaries, functional units, treatment of biogenic carbon, allocation procedures, electricity mixes, and assumptions regarding transportation and chemical recovery. As a result, apparently conflicting conclusions often reflect differences in assessment methodology rather than intrinsic differences in production technology.

Among the various production stages, activation is consistently identified as one of the most environmentally intensive operations because it simultaneously governs pore development, surface chemistry, adsorption capacity, and overall material performance while accounting for substantial energy and chemical consumption. The activation process largely determines the commercial value of activated carbon by controlling its specific surface area, pore-size distribution, and adsorption characteristics. However, maximizing these physicochemical properties frequently requires elevated operating temperatures, prolonged residence times, intensive chemical treatment, and extensive post-treatment operations, all of which increase energy demand, water consumption, and greenhouse gas emissions. Consequently, activation represents one of the clearest examples of the trade-off between material performance and environmental sustainability, where improvements in adsorption efficiency are often achieved at the expense of greater resource consumption.^49^

Chemical activation using potassium hydroxide (KOH) exemplifies this performance-sustainability dilemma. Owing to its exceptional ability to generate ultrahigh surface areas and highly developed microporous structures, KOH remains one of the most widely employed activating agents for producing activated carbons intended for advanced applications such as supercapacitors, gas separation, hydrogen storage, and water purification. Nevertheless, the environmental implications of this process extend beyond the activation step itself. The production of KOH is energy intensive, while its application requires substantial chemical inputs, repeated washing, large volumes of process water, and wastewater treatment to remove residual alkali. Accordingly, the environmental burden associated with KOH activation reflects not only thermal energy consumption but also the embodied impacts of chemical manufacture, chemical recovery, and effluent management.^50^

The importance of evaluating activation using a life-cycle perspective was clearly demonstrated by Saleem *et al.* (2025),^48^ who compared KOH- and NaOH-activated carbons produced from coconut shells using both mass-based and adsorption-based functional units. Their study showed that the KOH pathway required slightly greater energy input (28.31 MJ kg^−1^) than the NaOH route (27.06 MJ kg^−1^) and generated marginally higher climate change impacts (1.255 *versus* 1.209 kg CO_2_-eq kg^−1^). More importantly, contribution analysis identified pyrolysis and activation as the dominant environmental hotspots, while washing and chemical recovery further increased water use and secondary environmental burdens. However, the study also demonstrated that when activated carbon performance was evaluated on the basis of adsorption capacity rather than product mass, the superior adsorption efficiency of KOH-activated carbon compensated for its higher production impacts, improving overall functional environmental performance by approximately 6%. These findings emphasize that environmental assessments based solely on production emissions may underestimate the long-term sustainability of high-performance activated carbons, particularly where enhanced adsorption efficiency reduces material consumption during practical application. This distinction between mass-based and functional environmental assessment remains insufficiently considered in many published LCA studies and represents an important methodological limitation within the current literature.

Similar conclusions were reached by Li *et al.* (2025),^49^ who integrated life-cycle assessment, life-cycle costing, and energy-efficiency analysis to compare five commercial activated carbon production technologies. Their study demonstrated that the environmental performance of activated carbon depends not only on the activating agent but also on the overall integration of the production system. Steam-activated kernel shell-derived activated carbon exhibited the lowest overall environmental burden, representing only 13.28% of the impact associated with conventional anthracite-derived activated carbon. In contrast, sulfuric acid- and phosphoric acid-based activation routes generated substantially higher environmental impacts owing to increased sulfur dioxide emissions, greater industrial water consumption, and the embodied impacts associated with chemical manufacture. Furthermore, recovering carbonization by-products such as wood gas, wood tar, and wood vinegar for process energy increased energy recovery efficiency to 86.85% while reducing the overall energy conservation and emission reduction index by 45.31–67.19%. These observations demonstrate that environmental performance depends not only on activation chemistry but also on the degree of process integration, resource recovery, and energy reutilization incorporated into the production system. Consequently, future process optimization should focus on integrating waste heat recovery, by-product utilization, and chemical recycling rather than simply replacing one activating agent with another.

In contrast to chemical activation, physical activation using steam or carbon dioxide is generally considered to impose a lower environmental burden because it eliminates the need for corrosive activating chemicals and substantially reduces wastewater generation. From a life-cycle perspective, the absence of chemical reagents lowers embodied emissions associated with chemical manufacture while simplifying downstream effluent treatment. However, the environmental superiority of physical activation should not be generalized. The higher temperatures (typically 800–1000 °C) and longer activation times required for steam or CO_2_ activation substantially increase thermal energy demand, potentially offsetting the environmental benefits achieved through reduced chemical consumption. Moreover, physically activated carbons frequently exhibit lower surface areas and less-developed microporous structures than KOH-activated materials, particularly when low-reactivity biomass precursors are employed. Consequently, the choice between chemical and physical activation should not be based solely on production emissions but should instead consider adsorption efficiency, process energy, feedstock characteristics, intended application, and the carbon intensity of the energy supply. Rather than identifying one technology as universally superior, current evidence indicates that the most sustainable activation strategy is context-specific and depends on balancing physicochemical performance with life-cycle environmental impacts.

The comparative advantages and limitations of the principal activation technologies are summarized in Table 7, which illustrates the inherent trade-off between adsorption performance and environmental sustainability. As demonstrated by recent comparative LCAs, production routes yielding the highest-performance activated carbons are not necessarily those associated with the lowest environmental impacts. Accordingly, technology selection should be based on a holistic evaluation of adsorption efficiency, carbon yield, chemical consumption, energy demand, wastewater generation, and opportunities for resource recovery rather than on individual performance indicators alone.

**Table 7:** Comparison of environmental hotspots associated with major activation technologies^48–52^

Activation technology	Adsorption performance	Environmental burden	Principal emission sources	Sustainability assessment
KOH activation	Excellent (very high surface area and microporosity)	Very high	KOH manufacture, high-temperature heating, extensive washing, wastewater treatment	Highest adsorption performance but greatest environmental burden
H_3_PO_4_ activation	Excellent	High	Chemical production, washing, wastewater	High-performance process requiring careful waste management
ZnCl_2_ activation	Excellent	Very high	Zinc production, wastewater contamination	Effective but environmentally restricted in many jurisdictions
Steam activation	Moderate-high	Moderate	Thermal energy demand	Cleaner process with lower chemical consumption
CO_2_ activation	Moderate	Moderate	High-temperature gasification	Environmentally favourable but relatively energy intensive
Microwave-assisted activation	High	Low-moderate	Electricity consumption	Improved energy efficiency with reduced processing time
Hydrothermal carbonization + activation	Moderate-high	Low	Low-temperature processing; subsequent activation	Promising sustainable technology for wet biomass

Beyond the activation process, transportation represents another important environmental hotspot whose contribution is highly dependent on biomass characteristics, geographical location, and supply-chain configuration. Unlike fossil-derived precursors, most lignocellulosic biomass resources possess low bulk densities and are widely dispersed, requiring relatively large transportation volumes to satisfy industrial production demands. Consequently, transportation emissions increase disproportionately with haulage distance and become particularly significant where biomass is collected from multiple decentralized sources prior to processing. However, transportation should not be regarded as an inherently dominant environmental burden because its relative contribution varies considerably with regional logistics, biomass availability, and processing scale. Comparative life-cycle assessments have shown that transportation often contributes only a minor fraction of total greenhouse gas emissions when activated carbon production facilities are located close to biomass generation sites, whereas its environmental significance increases substantially in centralized production systems dependent on long-distance feedstock transportation. These observations suggest that optimizing supply-chain logistics and promoting decentralized processing facilities may provide environmental benefits comparable to those achieved through improvements in production technology.

The influence of transportation on environmental performance has been clearly illustrated by Vilén *et al.* (2022),^50^ who performed a comparative cradle-to-gate life-cycle assessment of activated carbon produced from coal, peat, coconut shells, wood, and reactivated activated carbon. Their study demonstrated that environmental impacts varied considerably with feedstock origin, transportation distance, and electricity generation, emphasizing that upstream logistics can significantly influence the overall carbon footprint of activated carbon production. More importantly, the study showed that reactivated activated carbon consistently exhibited the lowest environmental impacts among all evaluated production routes, illustrating that extending the service life of existing adsorbents may offer greater environmental benefits than continuously manufacturing virgin activated carbon from new biomass resources. This finding challenges the conventional emphasis on feedstock substitution alone and highlights regeneration as an underutilized strategy for reducing cumulative greenhouse gas emissions and resource consumption. Consequently, future environmental assessments should consider regeneration not merely as an end-of-life management option but as an integral component of sustainable activated carbon production systems.

Feedstock drying constitutes another major environmental hotspot, particularly for biomass resources characterized by high moisture contents, including sewage sludge, food waste, digestate, and several agro-industrial residues. Drying is essential for improving thermal efficiency, facilitating carbonization, and ensuring stable reactor operation; however, the process often requires substantial thermal energy that may significantly increase production-related greenhouse gas emissions. Importantly, the environmental burden associated with drying is determined not only by moisture content but also by the source of thermal energy employed. Conventional fossil fuel-based drying systems generally exhibit considerably higher carbon footprints than processes utilizing solar energy, waste heat, or biomass-derived process gases. Consequently, identical feedstocks may display markedly different environmental performances depending on the energy infrastructure supporting the drying process. This dependence underscores the importance of integrating waste heat recovery, renewable thermal energy, and energy-efficient drying technologies into future activated carbon manufacturing systems.

Electricity consumption similarly represents a critical environmental hotspot because virtually every production stage—including feedstock preparation, grinding, carbonization, activation, washing, drying, and post-treatment—depends directly or indirectly on electrical energy. Nevertheless, electricity should not be viewed as a fixed environmental burden because its contribution is governed primarily by the carbon intensity of the regional electricity grid rather than by activated carbon production itself. Manufacturing systems powered predominantly by coal-derived electricity generally exhibit substantially greater greenhouse gas emissions than equivalent facilities supplied by renewable energy, hydropower, or nuclear electricity. Accordingly, discrepancies among published life-cycle assessments frequently reflect regional differences in electricity generation rather than differences in production technology. This observation reinforces the importance of using geographically representative inventory data instead of generalized emission factors when comparing the environmental performance of activated carbon production systems across different countries.

The importance of electricity generation and energy supply was further demonstrated by Arena *et al.* (2016)^51^ in their life-cycle assessment of coconut shell-derived activated carbon. Their study identified electricity generation and thermal energy consumption among the principal contributors to global warming potential and demonstrated that replacing conventional electricity with renewable energy substantially reduced greenhouse gas emissions together with associated impacts such as fossil resource depletion and human toxicity. These findings indicate that improving the carbon intensity of energy supply may produce greater environmental benefits than optimizing individual unit operations alone. Similarly, comparative analyses consistently show that integrating renewable electricity with efficient thermal management strategies—including waste heat recovery, improved reactor insulation, and utilization of carbonization gases—offers one of the most effective pathways for reducing the life-cycle environmental burden of activated carbon production.

Recent comparative assessments have also highlighted the importance of evaluating environmental hotspots within an integrated systems framework rather than considering individual processes in isolation. Amin *et al.* (2025)^52^ compared activated carbon production from Fiddlewood (*Citharexylum spinosum*) and Jacaranda (*Jacaranda mimosifolia*) biomass using life-cycle assessment and demonstrated that overall environmental performance was governed by the combined influence of feedstock characteristics, activation conditions, process efficiency, and energy consumption rather than by any single production stage. Their findings reinforce the growing consensus that environmental sustainability is an emergent property of the entire production system, where interactions among biomass composition, thermal processing, electricity supply, transportation, and resource recovery collectively determine the final environmental footprint. Such observations challenge the common practice of attributing environmental impacts exclusively to activation or carbonization and instead support adopting a holistic systems-based perspective for environmental optimization.

Despite substantial progress in identifying environmental hotspots, important methodological limitations remain within the current body of literature. Many published life-cycle assessments employ different functional units, system boundaries, allocation procedures, electricity inventories, and assumptions regarding biogenic carbon sequestration, making direct comparison among studies inherently difficult. For example, some investigations express environmental performance per kilogram of activated carbon produced, whereas others normalize impacts according to adsorption capacity or pollutant removal efficiency. Similarly, transportation distances, activation yields, regeneration scenarios, and treatment of biomass-derived carbon differ considerably among studies, often leading to contrasting conclusions regarding the relative environmental performance of different feedstocks and activation technologies. Consequently, apparent inconsistencies among published studies frequently reflect methodological variation rather than genuine technological differences. Future comparative assessments would therefore benefit from greater harmonization of life-cycle methodologies, standardized functional units based on adsorption performance, and improved transparency in inventory reporting to facilitate robust comparison across different production systems.

Addressing these hotspots requires integrated process optimization rather than isolated unit-operation improvements. Local biomass sourcing, renewable energy, waste-heat recovery, and chemical recovery can reduce transport emissions, energy demand, and wastewater burdens. Optimization of activation conditions can further improve carbon yield and process efficiency, while regeneration and reuse of spent activated carbon can reduce cumulative life-cycle emissions compared with repeated production of virgin adsorbents.

## 5 Comparative life cycle assessment of different biomass feedstocks

The environmental sustainability of biomass-derived activated carbon is governed not simply by the renewable nature of the precursor, but by the interaction between feedstock composition, processing requirements, activation strategy, transportation logistics, and regional energy systems. Consequently, the environmental performance of a feedstock cannot be inferred from its biomass origin alone, because the same precursor may generate markedly different life-cycle impacts under different production conditions and system boundaries. This comparison is summarized in Table 8, which highlights the contrasting environmental and process characteristics reported for major biomass-derived activated carbon feedstocks. Comparative life-cycle assessments indicate that feedstocks requiring minimal pretreatment, providing relatively high carbon yields, and originating from existing waste streams can offer environmental advantages over dedicated raw materials requiring additional cultivation or intensive processing.^53–55^ However, these advantages are conditional rather than inherent, as differences in functional units, allocation procedures, electricity mix, transportation assumptions, and treatment of biogenic carbon can substantially alter the outcome of an LCA.

**Table 8:** Comparative environmental performance of biomass feedstocks used for activated carbon production^31,51,53–55^

Feedstock	Carbon footprint	Energy demand	Water footprint	Acidification	Human toxicity	Land use	Overall sustainability
Rice husk	Low	Moderate	Low	Low	Low	Very low	Excellent
Coconut shell	Low	Moderate	Low	Low	Low	Very low	Excellent
Palm kernel shell	Moderate	Moderate	Low	Low	Low	Low	Very good
Cassava peel	Low	Low	Low	Low	Low	Very low	Excellent
Corn cob	Low	Moderate	Low	Low	Low	Very low	Excellent
Coffee waste	Moderate	Moderate	Moderate	Moderate	Low	Very low	Good
Animal bone	Moderate	High	Moderate	Moderate	Moderate	Low	Good
Sewage sludge	High	High	High	High	High	Very low	Moderate

Agricultural residues are widely investigated because they combine existing waste streams with renewable carbon resources. Their environmental value therefore depends less on biomass availability than on whether their conversion avoids burdens associated with cultivation, disposal, and additional preprocessing. Differences in lignocellulosic composition, ash content, moisture, and carbon yield further determine the processing intensity and resulting environmental burden of each precursor.

Rice husk provides a useful example of this trade-off. Alade *et al.* (2012)^53^ compared activated carbon production from rice husk, milk bush kernel shell, and flamboyant pod bark using phosphoric acid activation and found substantial differences in environmental burdens among the precursors. Rice husk generated comparatively higher liquid and gaseous emissions, which the authors associated with its relatively high silica and ash content, although recovery of solid residues as secondary fuel reduced final waste disposal. Thus, rice husk cannot be considered environmentally superior merely because it is an agricultural residue; its inorganic composition can introduce additional processing burdens that partially offset the benefits of waste valorization.^53^

The performance dimension further complicates feedstock comparison. Oloriegbe *et al.* (2023)^54^ evaluated activated carbons derived from rice husk, maize cob, and coconut shell under identical carbonization and zinc chloride activation conditions and reported the highest adsorption performance for coconut shell, followed by maize cob and rice husk. Although this was not an LCA study, the findings demonstrate that precursor-dependent differences in material performance can influence sustainability at the application stage. A feedstock producing a more effective adsorbent may require less material to achieve a given treatment objective, meaning that production impacts should not be evaluated independently of functional performance.^54^

Coconut shell provides stronger evidence that process conditions can outweigh the environmental advantages attributed to feedstock type. Arena *et al.* (2016)^51^ identified electricity generation and thermal processing as major contributors to the global warming potential and human toxicity of coconut shell-derived activated carbon. Replacing conventional electricity with renewable biomass-derived energy reduced global warming impacts by approximately 80% and human toxicity by up to 60%, while production in regions supplied by low-carbon electricity further improved environmental performance. The implication is important for feedstock selection: even a favorable waste-derived precursor cannot guarantee a low-carbon product when energy-intensive processing is powered by carbon-intensive electricity.^51^

This conclusion is reinforced by the broader comparison of Lee and He (2025),^55^ who integrated adsorption measurements, thermodynamic modelling, LCA, and techno-economic analysis for activated carbons produced from sawdust, *Arundo donax*, Juncus grass, municipal solid waste, coconut shell, and palm kernel shell. KOH activation generated the highest CO_2_ adsorption capacities but also imposed greater environmental burdens because of increased chemical and energy requirements. Conversely, renewable electricity reduced life-cycle emissions by as much as 72%, and all evaluated biomass-derived activated carbons offset their production-related greenhouse gas emissions within 1–4 days of operation in a vacuum pressure swing adsorption system. Coconut shell and palm kernel shell provided particularly favorable combinations of adsorption performance, environmental performance, and economic viability. These results indicate that the environmental ranking of feedstocks is inseparable from activation chemistry, energy supply, and the performance achieved during use; therefore, “best” feedstock selection cannot be reduced to the precursor with the lowest production footprint.^55^

Palm kernel shell similarly illustrates the importance of system-level assessment. Its status as a by-product of palm oil processing provides an important waste-valorization advantage, while its lignocellulosic composition supports relatively high carbon yields and structural stability. However, these advantages do not establish a universally lower life-cycle burden because activation chemistry, transportation requirements, electricity generation, and process-chemical recovery can substantially modify its environmental profile. Comparative assessment should therefore treat palm kernel shell as a context-dependent option rather than inherently sustainable.

Evidence for corn cob remains less conclusive. He *et al.* (2023)^31^ demonstrated that microwave-assisted vacuum pyrolysis produced corn cob-derived activated carbon with a surface area approaching 995 m^2^ g^−1^, a high iodine number, and a relatively homogeneous pore structure while reducing processing time. These characteristics suggest potential process-efficiency advantages, but they cannot by themselves be translated into lower environmental impacts because the study did not quantify the corresponding life-cycle burdens. Accordingly, the available evidence does not justify ranking corn cob environmentally above other agricultural residues.^31^

The evidence challenges a simple feedstock-based hierarchy of environmental sustainability. Agricultural residues can provide important advantages through waste valorization and avoidance of dedicated cultivation, but the magnitude of these benefits is strongly conditioned by activation chemistry, energy source, transport, processing efficiency, and material performance (Table 8). The contrast between rice husk, coconut shell, palm kernel shell, and corn cob further demonstrates that precursor selection and process design should be evaluated as an integrated system rather than as independent variables. Moreover, the literature remains disproportionately focused on a limited number of feedstocks, particularly coconut shell and rice husk, while life-cycle evidence for cassava peel, corn cob, and other emerging residues remains comparatively limited. Future studies should therefore prioritize standardized comparative LCAs using consistent system boundaries, harmonized inventory data, and functional units linked to adsorption performance rather than production mass alone. Such an approach would provide a more defensible basis for determining whether a biomass-derived activated carbon pathway is genuinely environmentally preferable and under what technological and regional conditions that advantage is maintained.

## 6 Applications supporting carbon-negative technologies

The versatility of biomass-derived activated carbon extends far beyond its conventional role as an adsorbent. Owing to its high specific surface area, tunable pore architecture, chemical stability, and renewable origin, biomass-derived activated carbon has become an important functional material in numerous technologies that support carbon reduction, resource efficiency, and sustainable manufacturing. These applications not only enhance environmental protection but also contribute directly or indirectly to carbon-negative strategies by facilitating pollutant removal, renewable energy storage, greenhouse gas capture, and waste valorization.^32,43^

### 6.1 Water purification

Water purification is one of the most established applications of activated carbon, but the sustainability of biomass-derived alternatives cannot be inferred from their adsorption capacity alone. Their environmental advantage depends on whether the performance achieved during treatment compensates for impacts associated with precursor preparation, carbonization, activation, regeneration, and eventual disposal. Biomass-derived activated carbons have been investigated for dyes, pharmaceuticals, pesticides, metals, natural organic matter, endocrine-disrupting compounds, and PFAS, with regeneration offering an additional opportunity to reduce material demand and associated life-cycle burdens.^43,56–58^ The critical issue is therefore not whether biomass-derived activated carbon can adsorb contaminants, but whether its adsorption performance, regeneration stability, and production burden collectively provide a meaningful advantage over conventional activated carbon.

The available evidence further indicates that adsorption performance is controlled more directly by the structure and surface chemistry generated during processing than by biomass origin itself. Differences in lignin, ash, carbon yield, and mineral composition influence carbon formation, while activation conditions determine pore development and surface functionality. Thus, feedstock selection alone provides an inadequate basis for predicting treatment performance; the relevant comparison is between precursor-activation combinations and the functions they deliver under equivalent treatment conditions.^57^

This processing dependence is evident in the study of Hamid *et al.* (2025),^32^ who produced activated carbon from teak sawdust using ZnCl_2_ and NaOH activation. NaOH activation at 380 °C produced the highest iodine number (1124 mg g^−1^), whereas ZnCl_2_ generated larger and more interconnected pores. The contrasting structural characteristics produced by the two activating agents demonstrate that maximizing a single adsorption indicator does not necessarily identify the most suitable material for water treatment. Rather, activation must be matched to the contaminant and the transport requirements of the treatment process.

Yue *et al.* (2023)^43^ provide further evidence of the importance of tailoring pore structure to contaminant characteristics. Hydrothermal carbonization followed by alkaline activation of highland barley straw produced a mesoporous carbon with a specific surface area of 1906 m^2^ g^−1^, achieving 90.3% Cr(vi) removal within eight hours and 87.7% methylene blue removal within four hours. The ability to remove both inorganic and organic contaminants is significant, but the different removal rates also indicate that high surface area does not translate into uniform adsorption behaviour across contaminant classes. The study therefore supports the use of biomass-derived carbon as a versatile adsorbent, while also illustrating why material performance should be evaluated against contaminant-specific mechanisms rather than surface area alone.^43^

The practical relevance of biomass-derived activated carbon is more strongly demonstrated when multiple contaminants are considered simultaneously. Sherugar *et al.* (2021)^56^ developed activated carbon cartridges from *Rhizophora mucronata* mangrove fruit that removed heavy metals and dyes at efficiencies exceeding 99% within 12 min, with a specific surface area of 1061.5 m^2^ g^−1^. Compared with conventional single-solute batch experiments, the use of mixed wastewater provides stronger evidence of application potential; nevertheless, the study does not by itself establish how the adsorbent would perform under prolonged continuous-flow operation or repeated regeneration. This distinction is important because rapid removal under controlled conditions may not translate directly into sustained treatment performance.^56^

Heavy-metal adsorption likewise demonstrates the importance of surface chemistry. Queiroz *et al.* (2020)^57^ produced açaí-seed-derived activated carbon with surface areas of 1462–2774 m^2^ g^−1^, while nitric-acid modification increased oxygen-containing surface functionalities. The modified material achieved removal efficiencies of 86% for Pb^2+^ and 69% for Fe^2+^, with measurable Mg^2+^ adsorption exceeding that of commercial activated carbon. The comparison indicates that pore development alone is insufficient to explain adsorption selectivity: chemical modification can alter surface affinity and thereby determine which contaminants are preferentially retained. This supports a shift from surface-area-driven material development toward application-specific control of surface chemistry.^57^

The potential of biomass-derived activated carbon also extends to persistent emerging contaminants. Clouse *et al.* (2025)^58^ evaluated carbons prepared from lignin, cellulose nanocrystals, and cellulose nanofibers for regulated and unregulated PFAS. Adsorption efficiencies ranged from 31 to 98%, with the formulation containing 50% lignin, 35% cellulose nanocrystals, and 15% cellulose nanofibers removing more than 98% of seven PFAS compounds without additional surface functionalization. This finding is particularly relevant because it shifts the emphasis from simply converting waste biomass into activated carbon toward deliberate precursor engineering to achieve contaminant-specific affinity. However, the reported performance still requires validation under realistic water matrices, where competing organic matter and co-contaminants may alter PFAS adsorption.^58^

These studies challenge the assumption that adsorption capacity or specific surface area can serve as sufficient indicators of the suitability of biomass-derived activated carbon for water purification. Reported performance varies with precursor chemistry, activation route, pore architecture, surface functionality, contaminant concentration, pH, adsorbent dosage, and contact time.^43,56–58^ More importantly, much of the available evidence remains derived from batch experiments using individual contaminants. Such conditions simplify the adsorption environment and can overstate performance relative to real wastewater, where competition for adsorption sites, dissolved organic matter, variable ionic strength, and fluctuating contaminant concentrations are likely to influence removal.

The principal limitation is therefore not a lack of evidence for adsorption, but a lack of comparable and application-relevant evidence. High removal percentages obtained under optimized laboratory conditions cannot, without further validation, establish superiority over commercial activated carbon at treatment scale. Long-term regeneration, adsorbent stability, continuous-flow behaviour, contaminant competition, and the environmental burden of activation chemicals remain insufficiently integrated into performance assessments.

Future research should consequently prioritize performance-based rather than surface-area-based material development. Biomass-derived activated carbons should be evaluated using standardized contaminant mixtures and realistic water matrices under continuous-flow conditions, with simultaneous assessment of regeneration efficiency, service life, chemical consumption, energy demand, and life-cycle impacts. Coupling adsorption performance with techno-economic and life-cycle assessment would allow the environmental benefit of biomass-derived activated carbon to be judged on the basis of treatment delivered rather than adsorption capacity alone. This approach would provide a more robust basis for determining whether specific biomass-activation combinations can genuinely outperform conventional adsorbents and support their transition from laboratory-scale materials to sustainable water-treatment technologies.

### 6.2 Carbon dioxide capture

Carbon dioxide (CO_2_) capture has emerged as a promising application of biomass-derived activated carbon because it links greenhouse-gas mitigation with biomass waste valorization. Compared with conventional amine-based absorption, adsorption-based capture can offer lower regeneration energy requirements, good chemical stability, rapid adsorption–desorption kinetics, and repeated cycling, although these advantages depend strongly on adsorbent properties and operating conditions.^59^ The renewable origin of the precursor further supports the use of biomass-derived adsorbents for post-combustion capture, pressure swing adsorption (PSA), vacuum pressure swing adsorption (VPSA), and potentially negative-emission systems.^60^ However, the environmental advantage of these materials cannot be inferred from feedstock origin alone because activation chemicals, energy demand, washing requirements, and regeneration can substantially influence their life-cycle performance.

The central material-design challenge is therefore to establish the structural and chemical characteristics that maximize CO_2_ uptake under realistic operating conditions. Microporosity, particularly ultramicropores (<0.7 nm), can provide stronger confinement of CO_2_, while nitrogen-containing and other basic surface functionalities may enhance adsorbate–surface interactions. Surface area should therefore be treated as an incomplete performance indicator because adsorption also depends on pore-size distribution and surface chemistry. The available evidence supports application-specific control of pore architecture and surface functionality rather than generalized maximization of surface area.^59,60^

Cruz Junior *et al.* (2023)^59^ provide experimental evidence for this structure–performance relationship. Their KOH-activated cupuaçu-shell carbons exhibited specific surface areas of 1132–2486 m^2^ g^−1^ and micropore volumes of 0.73–1.02 cm^3^ g^−1^, with the optimized material achieving CO_2_ adsorption capacities of 7.8 mmol g^−1^ at 0 °C and 4.4 mmol g^−1^ at 25 °C. The relatively favorable isosteric heats of adsorption indicated predominantly physisorption, suggesting potentially lower regeneration energy requirements than strongly chemisorptive systems. Nevertheless, these results were obtained under controlled laboratory conditions; high adsorption capacity alone cannot establish industrial superiority, where moisture, competing gases, mass-transfer limitations, and cyclic operation are important.

Al-Ghurabi *et al.* (2025)^60^ similarly demonstrated that pyrolysis temperature, residence time, and KOH impregnation ratio substantially affected CO_2_ capture by date-palm-leaflet-derived activated carbon. The optimized material achieved 6.71 mmol g^−1^ at 0 °C and 4.214 mmol g^−1^ at 25 °C while maintaining performance during repeated adsorption–desorption cycles. The significance of this study lies in demonstrating that process variables can be manipulated to balance pore development and surface functionality. However, adsorption optimization does not necessarily minimize chemical consumption, energy demand, wastewater generation, or life-cycle emissions.

This trade-off is particularly evident for KOH activation. Although KOH activation can generate highly microporous carbons with excellent CO_2_ uptake, energy-intensive activation, chemical requirements, and post-activation washing can increase the environmental burden of production. For a carbon-footprint-focused assessment, therefore, adsorption capacity should be considered together with the environmental burden associated with producing and regenerating the adsorbent.

Another important limitation concerns the conditions under which adsorption performance is reported. Many laboratory studies use pure or highly concentrated CO_2_ streams, whereas industrial flue gas contains substantial N_2_, H_2_O, O_2_, NO_*x*_, and SO_*x*_, which can compete for adsorption sites, alter surface chemistry, or accelerate adsorbent degradation. Accordingly, adsorption capacities obtained under idealized conditions should be interpreted primarily as material-screening results rather than direct indicators of industrial capture performance. Dynamic fixed-bed measurements, breakthrough curves, cyclic working capacity, humid-gas testing, and long-duration regeneration studies are therefore more informative for evaluating practical suitability than equilibrium capacity alone.

The available evidence indicates that biomass-derived activated carbon can achieve high CO_2_ uptake, but its environmental benefit depends on the balance between capture performance and the burdens associated with production and regeneration. Future assessments should therefore combine realistic flue-gas testing, cyclic working capacity, performance-normalized LCA, and process-scale techno-economic analysis to determine whether improvements in capture performance translate into genuine life-cycle environmental benefits.

### 6.3 Soil amendment and carbon sequestration

The application of biomass-derived carbon materials to agricultural soils offers a potential pathway for linking soil management with carbon sequestration, but its climate benefit should not be assumed solely from the persistence of carbonaceous material in soil. The relevant question is whether the carbon retained in soil, together with any changes in soil greenhouse-gas emissions and agricultural inputs, produces a net climate benefit over the material's complete life cycle. This distinction is particularly important for activated carbon, because its production may require substantially more energy and chemical inputs than conventional biochar. Thus, soil application should be evaluated as a coupled material-production and soil-management system rather than as an isolated sequestration strategy.

The agronomic effects of biomass-derived carbon are similarly controlled by material properties and soil conditions rather than carbon content alone. Porosity, surface functionality, mineral composition, stability, and particle characteristics can influence water retention, nutrient interactions, microbial habitat formation, and soil structure. However, these mechanisms do not necessarily translate into consistent field-scale benefits. The response depends on soil texture, moisture regime, nutrient status, application rate, climatic conditions, and microbial activity, making simple extrapolation from material characterization or short-term laboratory experiments unreliable.

Ogawa *et al.* (2006)^61^ provided an early systems-level perspective through the Carbon Sequestration by Forestation and Carbonization (CFC) framework, which integrated biomass carbonization, soil amendment, and land restoration. Case studies from Indonesia, Australia, and Japan indicated that carbonized biomass could form a substantial component of long-term carbon sinks. In the Australian case, biochar incorporation represented approximately 52.8% of the total carbon sink over 35 years. The significance of this work is therefore not merely the magnitude of carbon storage reported, but its demonstration that sequestration outcomes depend on the integration of biomass production, carbonization, land application, and carbon permanence. The study also highlighted the need for standardized quality criteria and monitoring before soil carbon storage can be reliably incorporated into carbon-credit systems.

Subsequent evidence indicates that the interaction between carbon materials and soil is considerably more complex than direct carbon storage. The same surface and pore characteristics that determine adsorption behaviour can alter water redistribution, nutrient retention, microbial colonization, and rhizosphere processes, meaning that agronomic and climate effects may occur simultaneously and sometimes in competing directions. Kacaribu *et al.* (2026)^62^ advanced this interpretation by treating biomass-derived activated carbon, biochar, carbon granules, and related materials as functional soil interfaces rather than passive carbon reservoirs. Their synthesis indicates that pore accessibility, surface chemistry, mineral composition, ageing, and deployment conditions jointly determine soil performance. Importantly, BET surface area and maximum adsorption capacity were identified as inadequate standalone predictors of field behaviour, because soil texture, ionic strength, moisture fluctuations, wet–dry cycling, and microbial activity can substantially modify material functionality.

Enebe *et al.* (2025)^63^ likewise reported evidence for reduced microbial carbon mineralization, enhanced aggregate formation, increased microbial diversity, and greater carbon stabilization following biochar application. These findings strengthen the case for considering indirect sequestration mechanisms in addition to the carbon physically retained in the amendment itself. However, the reported variability with feedstock, pyrolysis conditions, application rate, soil characteristics, and climate indicates that these effects cannot be generalized across agricultural systems. A reduction in carbon mineralization observed under one soil condition should therefore not be interpreted as evidence of a universally persistent carbon sink.

This context dependence is also evident in the synthesis by Oyebiyi *et al.* (2026).^64^ Their comparison of organic and engineered soil amendments indicates that carbon-rich materials can improve soil structure, water retention, and nutrient-use efficiency, but that the magnitude and persistence of carbon sequestration are strongly site dependent. The implication is that amendment selection should begin with the specific soil constraint and desired function, rather than with the assumption that a material possessing superior laboratory physicochemical properties will necessarily deliver superior sequestration in the field. This is particularly relevant to activated carbon, for which high-temperature activation and chemical treatment may improve functional properties while simultaneously increasing the environmental burden associated with production.

An important unresolved issue is therefore the distinction between carbon storage potential and net carbon-removal potential. A biomass-derived carbon material can contain stable carbon and remain in soil for extended periods, yet its overall climate benefit may be reduced by emissions from biomass collection, transportation, drying, carbonization, chemical activation, washing, and application. Conversely, improvements in nutrient-use efficiency or soil water management could generate additional indirect benefits by reducing fertilizer requirements or other agricultural emissions. The net outcome must consequently account for both the carbon retained in soil and the emissions avoided or generated across the production-application system.

The evidence base also remains constrained by the limited duration and scale of most experiments. Short-term laboratory and greenhouse studies can identify mechanisms, but they cannot adequately establish carbon permanence, cumulative greenhouse-gas effects, ageing behaviour, or changes in soil–plant–microbe interactions over multiple growing seasons. This limitation is especially important because ageing may progressively alter pore accessibility, surface functionality, mineral interactions, and carbon stability, potentially changing both agronomic performance and sequestration efficiency.

Future research should therefore prioritize long-term field experiments coupled with life-cycle assessment and soil-carbon modelling. Studies should quantify not only changes in soil organic carbon but also CO_2_, CH_4_, and N_2_O fluxes, crop response, nutrient-use efficiency, amendment persistence, and changes in material properties during ageing. Comparisons should be based on a common functional basis that relates the environmental burden of producing the carbon material to the net carbon retained or removed over its operational lifetime. This would allow activated carbon to be evaluated against biochar and other soil amendments on the basis of net climate performance rather than carbon content alone.

The literature supports biomass-derived carbon materials as promising multifunctional soil amendments, but the evidence is not yet sufficient to regard activated carbon application as inherently carbon-negative or universally beneficial. Ogawa *et al.* (2006)^61^ established the potential for carbonized biomass to contribute substantially to long-term soil carbon sinks, while more recent syntheses demonstrate that soil functionality and carbon persistence are strongly controlled by material–soil interactions.^62–64^ The major research gap is therefore not whether biomass carbon can persist in soil, but whether activated carbon production and application can deliver a demonstrable net climate benefit while maintaining agronomic value under site-specific field conditions. Resolving this question through standardized, long-term, performance-based LCA and field validation is essential for establishing the genuine carbon-sequestration potential of biomass-derived activated carbon.

### 6.4 Electrochemical energy storage

Biomass-derived activated carbon has attracted considerable interest as an electrode material for supercapacitors, lithium-ion batteries (LIBs), sodium-ion batteries (SIBs), potassium-ion batteries (PIBs), and hybrid ion capacitors.^65,66^ Its principal advantage in this context is the ability to convert structurally diverse biomass into carbon architectures whose pore networks and surface chemistry can be tailored for ion storage. However, the suitability of a biomass precursor cannot be inferred from its origin; electrochemical performance ultimately depends on how precursor chemistry is translated into accessible pores, conductive pathways, and electrochemically active surface sites.

The key structure–performance relationship involves the coordination of pore hierarchy and surface chemistry. Micropores provide charge-storage sites, whereas meso- and macropores facilitate electrolyte penetration and ion transport. Heteroatoms such as nitrogen, phosphorus, sulfur, and oxygen can further modify surface wettability and contribute to pseudocapacitive charge storage.^66,67^ The critical issue is therefore pore accessibility rather than total pore volume: excessive activation may generate high surface area while producing poorly connected structures, increasing structural defects, or reducing electrical conductivity. Consequently, electrode design requires a balance between ion-accessible porosity and efficient electronic transport rather than indiscriminate development of porosity.

Niu *et al.* (2020)^65^ demonstrated this principle using heteroatom-doped porous carbon nanosheets prepared from gelatin and phytic acid. The material delivered an energy density of 135.3 Wh kg^−1^, a power density of 16.1 kW kg^−1^, and retained 88.6% of its initial capacity after 8000 cycles in sodium-ion capacitors. First-principles calculations linked the performance to the combined effects of hierarchical porosity and heteroatom incorporation, which promoted sodium-ion diffusion and charge-storage kinetics. Rather than demonstrating that one precursor is inherently superior, this study shows that electrochemical behaviour can be deliberately engineered by coupling pore architecture with chemical functionality.

Wei *et al.* (2017)^66^ extended this concept from electrode development to device integration. Using kelp as the precursor for the electrolyte, separator, binder, and activated-carbon electrode, they constructed a biomass-derived solid-state supercapacitor. The activated carbon exhibited a specific surface area of 4425 m^2^ g^−1^, a pore volume of 3.22 cm^3^ g^−1^, and a specific capacitance of 277 F g^−1^, together with good rate capability and cycling stability. The significance of this study lies in demonstrating that biomass-derived carbon can participate in the design of an integrated storage device rather than functioning solely as an isolated electrode material. Nevertheless, the very high surface area should not be interpreted as a direct measure of device superiority because accessible surface area, electrode density, electrolyte transport, and cell configuration collectively determine practical performance.

These studies also expose a limitation in how biomass-derived electrodes are commonly evaluated. Reported improvements are frequently attributed to a single parameter—surface area, heteroatom content, or pore volume—although these variables are interdependent. Increasing activation severity, for example, may improve microporosity but simultaneously reduce carbon yield or compromise conductivity. Similarly, heteroatom incorporation may enhance surface reactivity but can introduce additional synthesis steps. The most meaningful comparison is therefore not which material possesses the highest individual property, but which architecture delivers the required electrochemical response without disproportionate processing complexity.

Translation to practical devices remains another major limitation. Many reported materials are evaluated using three-electrode configurations, low or unspecified active-material loadings, and laboratory electrolytes. Such conditions can obscure limitations that become important in full cells, including electrode density, ion transport, voltage-window constraints, internal resistance, and irreversible capacity losses. Consequently, exceptional gravimetric capacitance or energy density obtained under idealized conditions cannot be assumed to represent commercial-device performance. Comparisons are further complicated by differences in electrolyte composition, current density, electrode formulation, voltage range, and cycling protocols.^65–67^

An additional unresolved issue is the relationship between material optimization and manufacturing scalability. Strategies involving extensive chemical activation, heteroatom doping, nanostructuring, or multistep functionalization may produce superior laboratory electrodes but can increase reagent consumption, processing complexity, wastewater generation, and production cost. This creates a practical trade-off between electrochemical optimization and manufacturability that remains insufficiently addressed in much of the literature. For biomass-derived electrodes to provide a meaningful sustainability advantage, this trade-off must be evaluated alongside, rather than after, electrochemical performance.

Future research should therefore shift toward device-relevant material optimization. Priority should be given to full-cell testing at realistic active-material loadings, reporting both gravimetric and volumetric performance, and evaluating long-term cycling, rate capability, and electrolyte compatibility under application-relevant conditions. Material synthesis should also be assessed in terms of precursor-to-carbon yield, activation intensity, chemical requirements, and process complexity. Combining these parameters with techno-economic and life-cycle analyses would allow researchers to identify electrode architectures that achieve an appropriate balance between performance, manufacturability, and environmental burden.

The available evidence demonstrates that biomass-derived activated carbon can serve as a versatile platform for electrochemical energy storage.^65–67^ The principal scientific opportunity is no longer simply to produce highly porous carbon, but to establish predictable relationships between precursor composition, activation, pore accessibility, surface chemistry, and device-level performance. The major unresolved challenge is therefore scaling this structure-function optimization without sacrificing electrochemical performance or introducing excessive production burdens. Addressing this challenge will be essential for moving biomass-derived activated carbons from laboratory-scale electrode demonstrations toward technically and commercially credible energy-storage technologies.

### 6.5 Hydrogen storage

Hydrogen storage remains a major limitation in the development of hydrogen-based energy systems because practical storage requires high uptake together with reversibility, safety, and acceptable operating conditions. Biomass-derived activated carbon provides a porous carbon framework in which hydrogen is stored predominantly through reversible physisorption. Its principal advantage, therefore, lies not simply in its biomass origin but in the ability to tailor pore dimensions to strengthen hydrogen–carbon interactions without compromising reversibility.^67^

The importance of pore dimensions is demonstrated particularly clearly by Masika and Mokaya (2012).^67^ Using porous carbons with comparable specific surface areas, they showed that reducing the average pore width from 31 Å to 12 Å increased excess hydrogen uptake from 3.7 wt% to 6.3 wt% at 77 K and 20 bar, despite an identical surface area of approximately 3340 m^2^ g^−1^. This approximately 70% increase demonstrates that the spatial distribution of carbon surfaces is more informative for hydrogen storage than BET surface area considered independently. The result also indicates that appropriately narrow pores can generate stronger overlapping adsorption potentials, providing a structural basis for improving hydrogen uptake.^67^

Surface modification has subsequently been explored to strengthen hydrogen adsorption beyond the capability of porous carbon alone. Hwang *et al.* (2021)^68^ prepared activated porous carbon fibers with a BET surface area of 3058 m^2^ g^−1^ and a micropore volume of 1.18 cm^3^ g^−1^, achieving 5.14 wt% hydrogen uptake at 77 K and 10 MPa. Incorporation of palladium nanoparticles increased this value to 5.45 wt%, suggesting a contribution from hydrogen-spillover effects.^68^ However, the relatively limited increase in uptake indicates that the benefit of catalytic modification must be weighed against the additional material requirements and processing complexity. Thus, metal decoration should be regarded as a complementary strategy rather than a substitute for appropriate pore development.

The evidence consequently points to a pore-size-dependent storage mechanism in which ultramicropores provide favorable adsorption environments, while larger pores primarily contribute to molecular accessibility. This creates an important optimization constraint: excessive activation may enlarge pores or reduce carbon yield without producing a proportional increase in hydrogen uptake. The objective should therefore be selective development of pores within the size range most favorable for hydrogen adsorption rather than maximum porosity.

The major unresolved limitation is the operating temperature. The high capacities reported by Masika and Mokaya (2012)^67^ and Hwang *et al.* (2021)^68^ were obtained at 77 K, with Hwang *et al.* additionally using 10 MPa. Under ambient conditions, weaker hydrogen–carbon interactions substantially restrict physisorption. Consequently, reported cryogenic capacities provide evidence of material potential but cannot, by themselves, establish suitability for practical hydrogen-storage systems. Moreover, gravimetric uptake may overstate system-level performance where highly porous carbons have low bulk density; volumetric capacity therefore requires equal consideration.

Accordingly, the critical research challenge is not merely to produce biomass-derived carbons with higher hydrogen uptake, but to retain useful adsorption at less demanding temperatures while achieving adequate volumetric storage and reversible cycling. Future investigations should therefore report gravimetric and volumetric capacities together and evaluate adsorption under standardized temperature–pressure conditions. Particular attention should be given to establishing whether ultramicropore optimization can deliver meaningful improvements under near-ambient conditions and whether modifications such as palladium incorporation provide sufficient performance gains to justify their added complexity. These considerations are essential for determining the realistic technological potential of biomass-derived activated carbon for hydrogen storage.

### 6.6 Catalysis and catalyst supports

Biomass-derived activated carbon can function as more than a conventional catalyst carrier because its surface and pore environment can influence the dispersion, accessibility, and interaction of catalytically active species. This distinction is important: catalytic performance depends on the interaction between the carbon matrix and the active phase rather than on the intrinsic properties of either component considered independently. Such characteristics have supported applications in biodiesel production, photocatalysis, electrocatalysis, advanced oxidation, and environmental catalysis.^69,70^

Evidence from biodiesel production illustrates this support effect. Ali *et al.* (2024)^69^ prepared potassium-containing catalysts from malt bagasse using two preparation routes. Although catalyst C2 contained substantially more potassium, catalyst C1 achieved a biodiesel yield of 91.8% and retained 88.7% after reuse. The higher activity of C1 despite its lower potassium content indicates that active-phase accessibility and distribution can be more important than loading itself. Thus, increasing the quantity of an active component does not necessarily translate into improved catalytic conversion.^69^

Surface functionalization provides a different approach to modifying catalytic behaviour. Ao *et al.* (2024)^70^ developed sulfonated activated carbon (BT-SO_3_H) from banana trunk for the conversion of *Chlorella vulgaris* lipids to biodiesel. The material achieved a 58.29% biodiesel yield under optimized conditions. Unlike a support that merely immobilizes an active species, the sulfonated carbon contributed directly through its acidic functional groups, demonstrating that chemical modification can transform biomass-derived carbon into a catalytic material in its own right. This is particularly relevant to esterification and transesterification of lipid feedstocks containing substantial free fatty acids.^70^

The influence of the active phase is evident in the work of Borand *et al.* (2025),^71^ who supported Cu–Zn, Fe–Zn, and Cu–Fe bimetallic catalysts on spruce-sawdust-derived activated carbon. The resulting biodiesel yields were 89.02%, 74.67%, and 68.16%, respectively. Because the carbon support was common to all three systems, the markedly different activities highlight the importance of metal composition and metal–carbon interfacial interactions in determining catalytic output. The findings therefore argue against evaluating activated carbon supports independently of the catalytic phase they carry.^71^

A further direction is illustrated by He *et al.* (2025),^72^ who developed an S-scheme g-C_3_N_4_-TiO_2_@montmorillonite photocatalyst that achieved 98.5% biodiesel yield. Spectroscopic and density functional theory analyses showed that interfacial electric fields enhanced charge separation and carrier migration, promoted oleic-acid enrichment, and lowered the activation energy of the rate-determining step. Although activated carbon was not used in this system, the study provides a useful mechanistic benchmark for carbon-supported photocatalysts: controlling interfaces and local reaction environments may be more consequential than simply increasing catalyst loading. This principle offers a basis for designing biomass-derived carbon supports for photocatalytic and electrocatalytic applications.^72^

The combined evidence therefore suggests that the key value of biomass-derived activated carbon in catalysis lies in its capacity to regulate the interface between the reactant and active phase. However, the literature remains disproportionately focused on biodiesel production, with substantially less evidence for applications such as photocatalytic hydrogen production, oxygen reduction/evolution, CO_2_ reduction, and selective chemical synthesis. Moreover, many reported systems have been assessed only under batch laboratory conditions, leaving uncertainty regarding active-phase leaching, deactivation, regeneration, continuous operation, and long-term stability.^69–71^

An important unresolved issue is consequently the lack of transferable structure–interface–activity relationships. Future work should establish how precursor characteristics and carbon-processing conditions affect the interaction of activated carbon with specific catalytic phases and, in turn, reaction selectivity and stability. Rather than reporting catalytic yield alone, studies should distinguish the contribution of the carbon support from that of the active phase and evaluate performance over repeated cycles and under continuous operation. Such evidence would provide a stronger basis for determining whether biomass-derived activated carbon can serve as a genuinely functional component of scalable catalytic systems rather than simply as a low-cost support.

### 6.7 Air purification

Air purification represents one of the earliest and most commercially successful applications of activated carbon because of its exceptional ability to remove volatile organic compounds (VOCs), sulfur-containing gases, nitrogen oxides (NO_*x*_), odorous compounds, and a wide range of airborne contaminants through physical adsorption and surface-mediated reactions. Biomass-derived activated carbon has emerged as a sustainable alternative to conventional coal-derived activated carbon by providing comparable adsorption performance while simultaneously reducing reliance on fossil-derived precursors and promoting agricultural waste valorization. The combination of high specific surface area, hierarchical pore architecture, and abundant surface functional groups enables efficient adsorption of gaseous pollutants across domestic, industrial, and environmental applications. Consequently, biomass-derived activated carbon has become increasingly important in indoor air purification, industrial emission control, protective filtration, and environmental pollution mitigation.^72,73^

The efficiency of biomass-derived activated carbon in air purification depends not only on its specific surface area but also on the accessibility of micropores, pore-size distribution, surface chemistry, and the physicochemical characteristics of the target pollutants. Micropores predominantly govern adsorption of low-molecular-weight gases through pore-filling mechanisms, whereas mesopores facilitate rapid diffusion of larger volatile organic molecules toward adsorption sites. Surface oxygen-containing functional groups further influence adsorption selectivity by modifying interactions between pollutant molecules and the carbon surface. Accordingly, recent research has shifted from maximizing adsorption capacity alone toward engineering multifunctional adsorbents capable of combining pollutant capture with antimicrobial activity, catalytic oxidation, and regeneration capability.^73^

Recent experimental investigations demonstrate that biomass-derived carbon-based sorbents can effectively remove particulate pollutants while simultaneously providing biological protection. Mutushev *et al.* (2025)^73^ developed carbon–silicon sorbents that efficiently captured airborne particles ranging from 0.65 to 150 µm, achieving organic dust removal efficiencies between 74% and 98%. In addition to particulate removal, the optimized sorbent exhibited measurable antibacterial activity against *Bacillus paramycoides* and *Pseudomonas koreensis*, indicating that appropriately engineered carbon-based adsorbents can provide multifunctional air purification by combining particle filtration with microbial suppression. These findings are particularly significant because conventional activated carbon systems primarily target gaseous pollutants and VOCs but generally possess limited intrinsic antimicrobial activity. The incorporation of additional active components therefore broadens the functionality of biomass-derived carbon materials beyond adsorption alone, making them attractive for applications requiring improved indoor air hygiene, healthcare facilities, and clean-room environments.

An equally important challenge in air purification is the regeneration of spent activated carbon. Conventional adsorption systems gradually become saturated, requiring replacement or energy-intensive off-site regeneration, thereby increasing operational costs and environmental impacts. Addressing this limitation, Raso *et al.* (2014)^74^ developed an integrated two-stage system combining activated carbon adsorption with catalytic oxidation using a CeO_2_/TiO_2_ catalyst. Rather than discarding saturated activated carbon, the adsorbed VOCs were periodically desorbed through controlled thermal regeneration and subsequently oxidized within the catalytic unit. Experimental evaluation involving representative VOCs, including limonene, linalool, and *n*-decane, demonstrated efficient cyclic adsorption–desorption performance while significantly reducing maintenance requirements and improving energy efficiency. This study illustrates an important transition in air purification technology from simple pollutant capture toward integrated adsorption-catalysis systems capable of simultaneously improving pollutant removal, reducing secondary waste generation, and extending adsorbent service life.

These experimental studies demonstrate that the role of biomass-derived activated carbon in air purification extends beyond passive adsorption. Recent developments increasingly integrate adsorption with complementary functionalities, including catalytic oxidation, antimicrobial protection, and self-regeneration, thereby overcoming several limitations associated with conventional activated carbon filters. Such multifunctional systems not only improve pollutant removal efficiency but also reduce operational costs, increase adsorbent longevity, and minimize environmental impacts associated with spent adsorbent disposal. These developments are consistent with broader trends toward circular and sustainable air purification technologies.

Despite substantial progress, important research gaps remain. Most investigations continue to evaluate adsorption performance using individual pollutants under controlled laboratory conditions, whereas actual indoor and industrial atmospheres contain complex mixtures of VOCs, particulate matter, sulfur compounds, nitrogen oxides, moisture, and biological contaminants that may compete for adsorption sites or alter adsorption behaviour. Furthermore, relatively few studies have systematically examined long-term adsorption performance, regeneration efficiency over multiple operational cycles, mechanical durability, or the influence of fluctuating humidity and temperature on adsorption capacity. The absence of standardized testing protocols further complicates comparison among adsorbents prepared from different biomass precursors, activation methods, and operating conditions.

Future research should therefore focus on engineering multifunctional biomass-derived activated carbons possessing optimized hierarchical pore structures, tailored surface chemistry, and enhanced regeneration capability for simultaneous removal of gaseous pollutants, particulate matter, and airborne microorganisms. Greater attention should also be directed toward integrating activated carbon with photocatalysts, antimicrobial nanoparticles, and advanced oxidation technologies to produce self-regenerating air purification systems capable of continuous pollutant degradation rather than simple adsorption. In addition, coupling material development with life-cycle assessment, techno-economic evaluation, and pilot-scale validation will facilitate the transition of biomass-derived activated carbon from laboratory-scale studies to commercially viable air purification technologies capable of supporting cleaner indoor environments and sustainable industrial emission control.

## 7 Industrial-scale implementation

Although laboratory studies dominate the development of biomass-derived activated carbon, available evidence indicates that scale-up is technically feasible, although maturity varies substantially among production routes. Zhou *et al.* (2020)^75^ reported the commercial performance of a biomass-gasification system integrating clean heating and activated-carbon production, demonstrating that biomass conversion and activated-carbon manufacture can be integrated within a commercial process. This integration is important because large-scale feasibility depends not only on adsorbent quality but also on efficient process-energy utilization.

More direct evidence was provided by de la Torre *et al.* (2025),^76^ who evaluated physical activation of palm-kernel shell at pilot scale in a Nichols furnace and industrial scale in a rotary kiln. Their results demonstrated that the progressive-conversion model could describe activation behaviour at both scales, while H_2_O-rich activation produced greater porosity development and higher iodine indices than CO_2_ activation. The study therefore provides evidence that reaction kinetics and process modelling can support the transfer of laboratory activation processes to continuous industrial equipment.

Industrial-scale feasibility has also been examined through process simulation and techno-economic assessment. Karimi and Shirzad^77^ designed an industrial plant for producing CO_2_ adsorbent from municipal-solid-waste digestate, with a proposed capacity of 90 273 tonnes of MSW annually and approximately 4788 tonnes of activated-carbon adsorbent per year. The estimated net electricity and heat requirements were 1367.8 MWh and 20 134.4 GJ, respectively, with a production cost of approximately US$0.61 kg^−1^. However, this study represents an industrial process design rather than evidence from an operating full-scale plant and should therefore be interpreted as an indication of engineering and economic feasibility rather than established commercial deployment.

The available techno-economic evidence remains limited and heterogeneous. Production costs are strongly influenced by feedstock price, plant capacity, activation route, energy source, chemical consumption, product yield, and assumptions concerning coproduct revenues and waste-management credits. Furthermore, many laboratory- and pilot-scale studies do not report complete CAPEX, OPEX, profitability, payback, or sensitivity analyses. Consequently, current evidence demonstrates promising economic potential but remains insufficient to establish broad cost competitiveness across biomass feedstocks and production technologies.

From a carbon-footprint perspective, scale-up may also produce outcomes that differ from laboratory estimates. Larger systems may improve thermal integration and energy recovery but can simultaneously increase transportation requirements, process-energy demand, wastewater generation, and continuous-operation emissions. Industrial feasibility should therefore be evaluated together with life-cycle and techno-economic performance, particularly for chemically activated materials where reagent consumption, recovery, and wastewater treatment may become increasingly important at commercial production volumes.

Therefore, future research should prioritize long-duration continuous demonstrations, standardized scale-up protocols, process-energy integration, chemical recovery, product-quality control, and integrated LCA-TEA assessment. Such evidence is necessary to determine whether biomass-derived activated carbon can progress from promising laboratory materials to commercially competitive and demonstrably low-carbon industrial products.

## 8 Challenges

Despite significant advances in biomass-derived activated carbon, several scientific, technological, economic, and methodological challenges continue to limit its wider implementation and its potential to deliver genuine climate benefits. The principal challenges concern feedstock variability, LCA harmonization and data quality, resource requirements, regeneration and end-of-life management, economic feasibility, industrial scale-up, and verification of carbon-negative performance.

### 8.1 Feedstock variability

Biomass feedstocks vary substantially in cellulose, hemicellulose, lignin, ash, mineral composition, and moisture content as a result of species, geography, seasonality, agricultural practices, and storage conditions. This variability affects carbon yield, pore development, surface chemistry, and adsorption performance and complicates process optimization and industrial quality control. Standardized feedstock classification and adaptive process-control strategies are therefore required to improve reproducibility.

### 8.2 LCA methodological harmonization

Comparisons of environmental performance remain difficult because existing studies use different functional units, system boundaries, allocation procedures, electricity datasets, transportation assumptions, emission factors, and impact-assessment methods. Such differences can produce substantially different results for otherwise similar production pathways. Harmonized LCA frameworks specifically addressing biomass-derived activated carbon are therefore needed to improve comparability and reduce interpretation based on modelling choices.

### 8.3 Life-cycle inventory and data quality

Reliable assessment is further constrained by limited data on energy consumption, chemical production and recovery, water use, wastewater treatment, transportation, regeneration, and industrial-scale operation. Many studies rely on laboratory measurements, simulated processes, or generic databases that may not represent commercial conditions. Expanded industrial-scale inventories, transparent reporting, and geographically representative datasets are therefore necessary to reduce uncertainty in carbon-footprint assessments.

### 8.4 Water and wastewater from chemical activation

Chemical activation can generate substantial environmental burdens through activating-agent production, extensive washing, freshwater consumption, and treatment of acidic or alkaline wastewater. These impacts can offset some of the environmental benefits associated with waste-biomass utilization, particularly when high chemical loadings are required. Chemical recovery, water recycling, and closed-loop processing should therefore receive greater attention in future process development.

### 8.5 Regeneration and end-of-life

Although regeneration can extend activated-carbon service life and reduce demand for virgin material, repeated regeneration requires energy and may progressively alter adsorption performance. The environmental implications of multiple regeneration cycles and final disposal, recycling, or secondary use remain insufficiently quantified. Life-cycle assessments should therefore incorporate realistic service lifetimes, regeneration frequency and efficiency, and end-of-life carbon fate.

### 8.6 Economic feasibility

Commercial competitiveness remains constrained by costs associated with thermal energy, activating chemicals, washing, drying, transportation, quality control, and environmental-management infrastructure. Although waste biomass may have low feedstock costs, these additional burdens can strongly influence production economics. Integrated techno-economic and environmental assessments are therefore needed to identify pathways that simultaneously achieve economic viability and reduced environmental impacts.

### 8.7 Industrial-scale implementation

Evidence for commercial and industrial-scale production remains limited compared with the large body of laboratory research. Scale-up introduces challenges involving reactor design, heat and mass transfer, process control, product consistency, energy efficiency, and chemical recovery, while laboratory adsorption results may not adequately represent complex industrial operating conditions. Long-duration continuous demonstrations, standardized scale-up protocols, process-energy integration, chemical recovery, product-quality control, and integrated LCA-TEA assessment remain important priorities for establishing reliable commercial performance.

### 8.8 Carbon-negative verification and permanence

The most fundamental challenge is determining whether biomass-derived activated carbon can genuinely achieve a net-negative carbon balance rather than simply a lower carbon footprint than fossil-derived alternatives. This requires accounting for biomass sourcing and counterfactual fate, biogenic carbon, production and transport emissions, energy and chemical inputs, carbon stability and permanence, regeneration, and end-of-life pathways within consistent system boundaries. Carbon-negative claims should therefore be supported by transparent carbon accounting, explicit treatment of uncertainty and temporal carbon storage, and evidence that durable carbon retention or removal exceeds attributable life-cycle greenhouse-gas emissions.

## 9 Conclusions

Biomass-derived activated carbon offers a promising pathway for biomass valorization and potentially lower-carbon activated-carbon production; however, renewable biomass origin alone does not establish environmental sustainability or carbon-negative performance. The review establishes that life-cycle outcomes depend on the complete production and use pathway, including feedstock sourcing, energy and chemical inputs, activation, application, regeneration, and end-of-life management. A key finding is that published LCA results are strongly influenced by methodological choices, including system boundaries, functional units, energy mixes, allocation procedures, emission factors, and treatment of biogenic carbon. These differences limit direct comparison among studies and indicate that reported environmental advantages should be interpreted within their specific modelling assumptions. Carbon-negative performance requires a more stringent demonstration in which life-cycle greenhouse-gas emissions are evaluated against the quantity and durability of carbon retained or removed. In this context, the counterfactual fate of biomass, carbon permanence, regeneration, and end-of-life pathways are particularly important because waste-biomass utilization or carbon retention cannot automatically be interpreted as permanent atmospheric carbon removal. The evidence reviewed therefore supports a conditional rather than inherent carbon-negative potential for biomass-derived activated carbon. Some production pathways may achieve lower carbon footprints, and potentially net-negative carbon balances, under favourable conditions; however, the available evidence remains insufficient to generalize carbon negativity across feedstocks, activation technologies, applications, or geographical contexts. Future research should prioritize standardized and transparent LCA frameworks combined with techno-economic assessment and pilot- or industrial-scale validation. Such assessments should explicitly account for counterfactual biomass fate, biogenic carbon, carbon-storage duration, regeneration, end-of-life emissions, supply-chain impacts, and uncertainty. These improvements are necessary to determine whether environmental benefits observed at laboratory or modelled scales remain robust under realistic operating conditions. Biomass-derived activated carbon should be regarded as a potentially lower-carbon and, under appropriately demonstrated conditions, carbon-negative material rather than inherently carbon-negative. Its climate contribution will ultimately depend on the demonstrated balance between life-cycle emissions and durable carbon retention or removal across the defined system boundary.

## Conflicts of interest

The authors declare that they have no known competing financial interests or personal relationships that could have appeared to influence the work reported in this paper.

## Data Availability

No new data were generated or analyzed in this study. This article is based on a comprehensive review and synthesis of previously published literature. All data supporting the findings of this review are available within the cited references.

## References

[cit1] (a) Ritchie H. and Roser M., CO_2_ emissions, Our World in Data, 2020, https://ourworldindata.org/co2-emissions

[cit2] Onaiwu G. E., Ifijen I. H. (2024). PM2.5-bound polycyclic aromatic hydrocarbons (PAHs): Quantification and source prediction studies in the ambient air of automobile workshop using the molecular diagnostic ratio. Asian J. Atmos. Environ..

[cit3] Ndem P., Biose O., Effiong E. (2025). *et al.*, Electrochemical sensor-based assessment of CO_2_ and CH_4_ levels in Benin City under extreme heat wave conditions in Nigeria. Environ. Monit. Assess..

[cit4] Oghoje S. U., Omoruyi I. C., Ejeomo C. (2024). *et al.*, Enhancing petroleum-contaminated soil remediation using pulverized rice straw. Chem. Pap..

[cit5] Oghoje S. U., Omoruyi C. I., Ojeomo C. (2023). *et al.*, Revolutionizing soil remediation: Harnessing the potential of chicken manure digestates for petroleum hydrocarbon contamination. Chem. Afr..

[cit6] Badiger S., Nidheesh P. V. (2024). Coconut shell biochar for the removal of acetaminophen and ciprofloxacin in low concentrations: Single and competitive adsorption studies. Ind. Eng. Chem. Res..

[cit7] Aljubiri S. M., Younes A. A. O., Alosaimi E. H., Abdel Daiem M. M., Abdel-Salam E. T., El-Shwiniy W. H. (2024). Recycling of sewage sludge: Synthesis and application of sludge-based activated carbon in the efficient removal of cadmium (II) and lead (II) from wastewater. Int. J. Mol. Sci..

[cit8] Chopade G., Devatha C. P. (2024). Experimental investigation on sludge conditioning and dewatering using an agricultural biomass coupled with resource recovery. J. Environ. Manage..

[cit9] Chew T. W., H’ng P., Abdullah B. C. T. G. L. C., Chin K. L., Lee C. L., Hafizuddin B. M. S. M. N., TaungMai L. (2023). A review of bio-based activated carbon properties produced from different activating chemicals during chemical activation process on biomass and its potential for Malaysia. Materials.

[cit10] Gale M., Nguyen T., Moreno M., Gilliard-AbdulAziz K. L. (2021). Physiochemical properties of biochar and activated carbon from biomass residue: Influence of process conditions to adsorbent properties. ACS Omega.

[cit11] Hossen M. S., Islam T., Hasan M. Z., Bashar M. M., Luo L., Narita F. (2026). Adv. Sustainable Syst..

[cit12] Villora-Picó J. J., González-Arias J., Baena-Moreno F. M., Reina T. R. (2024). Materials.

[cit13] Manimekala T., Sivasubramanian R., Dar M. A., Dharmalingam G. (2025). RSC Adv..

[cit14] (a) Conradi-Galnares E. , Blandón-González B. and Marrero-Meléndez M., in Life Cycle Analysis Based on Nanoparticles Applied to the Construction Industry, ed. P. Mercader-Moyano and P. Porras-Pereira, Springer, 2025, pp. 23–44, https://dx.doi.org/10.1007/978-3-031-79115-4_2

[cit15] Mudersbach M., Jürgens M., Pohler M., Spierling S., Venkatachalam V., Endres H.-J., Barner L. (2025). Macromol. Rapid Commun..

[cit16] Finkbeiner M., Inaba A., Tan R., Klüppel H.-J., Christiansen K. (2006). Int. J. Life Cycle Assess..

[cit17] Liu M., Zhu G., Tian Y. (2024). Green Carbon.

[cit18] Bedia J., Peñas-Garzón M., Gómez-Avilés A., Rodriguez J. J., Belver C. (2020). C.

[cit19] Wang X., Cheng H., Ye G., Fan J., Yao F.-F., Wang Y., Jiao Y., Zhu W., Huang H., Ye D. (2022). Chemosphere.

[cit20] Scholz M. (2023). Technologies.

[cit21] Sharma G., Sharma S., Kumar A., Lai C., Naushad M., Iqbal S., Stadler F. (2022). Activated Carbon as Superadsorbent and Sustainable Material for Diverse Applications. Adsorpt. Sci. Technol..

[cit22] Serafin J., Dziejarski B. (2023). Activated carbons—preparation, characterization and their application in CO2 capture: A review. Environ. Sci. Pollut. Res..

[cit23] Zhang S., Zheng M., Tang Y., Zang R., Zhang X., Huang X., Chen Y., Yamauchi Y., Kaskel S., Pang H. (2022). Understanding Synthesis–Structure–Performance Correlations of Nanoarchitectured Activated Carbons for Electrochemical Applications and Carbon Capture. Adv. Funct. Mater..

[cit24] Gao Y., Yue Q., Gao B., Li A. (2020). Insight into activated carbon from different kinds of chemical activating agents: A review. Sci. Total Environ..

[cit25] Isaeva V. I., Vedenyapina M. D., Kurmysheva A. Y., Weichgrebe D., Nair R. R., Nguyen N. P. T., Kustov L. M. (2021). Modern carbon-based materials for adsorptive removal of organic and inorganic pollutants from water and wastewater. Molecules.

[cit26] David S., Kim S., Oh S., Kahng Y. H. (2025). Advances in decorated mobile composites for adsorption and photocatalytic dye degradation: A comprehensive review. J. Hazard. Mater. Adv..

[cit27] Rahman M. M., Ara M. G., Alim M. A., Uddin M. S., Najda A., Albadrani G. M., Sayed A. A., Mousa S. A., Abdel-Daim M. M. (2021). Mesoporous carbon: A versatile material for scientific applications. Int. J. Mol. Sci..

[cit28] Groen J. C., Peffer L. A. A., Pérez-Ramírez J. (2003). Pore size determination in modified micro- and mesoporous materials: Pitfalls and limitations in gas adsorption data analysis. Microporous Mesoporous Mater..

[cit29] Mane P. V., Rego R. M., Yap P. L., Losic D., Kurkuri M. D. (2024). Unveiling cutting-edge advances in high surface area porous materials for the efficient removal of toxic metal ions from water. Prog. Mater. Sci..

[cit30] Tewari S., Kaur J., Tandon S. (2026). *et al.*, Recent advances in functionalized nanocarbon adsorbents for toxic pollutant removal: Challenges and future perspectives. Discover Nano.

[cit31] He X., Chen X., Wang X., Jiang L. (2023). Optimization of activated carbon production from corn cob using response surface methodology. Front. Environ. Sci..

[cit32] Hamid S. G., Arief T., Anggamulia M. I., Azis H. A., Arman M. (2025). Activated Carbon from Sawdust Biomass Via ZnCl_2_ and NaOH Activation for Water Biofiltration Applications. J. Green Chem. Environ. Eng..

[cit33] Kinate B. B., Abinoye F. E., Dune K. K., Elikee U. D. (2025). Experimental investigation of the use of animal bone waste and corn husk activated carbon for oil and gas produced water treatment. Improv. Oil Gas Recovery.

[cit34] Yang P., Yu M., Ma X., Deng D. (2023). Carbon Footprint of the Pork Product Chain and Recent Advancements in Mitigation Strategies. Foods.

[cit35] Ben Salah A., Antxustegi M. M., Corro E., Volpe R., González-Alriols M. (2026). A competitive approach for the production of activated carbons from lignocellulosic biomass. Clean Technol. Environ. Policy.

[cit36] Carrejo E., Ortega R., Lan K., Sanchez D. L., Park S., Sagues W. J. (2026). Quantifying atmospheric carbon removal at pulp and paper mills: a life cycle assessment across system boundaries. Carbon Neutrality.

[cit37] Cambazoğlu M., Coşkun S., Sütçü A. (2025). Carbon footprint evaluation of melamine-coated particleboard production: A case study in Türkiye. BioResources.

[cit38] Kennelly C., Berners-Lee M., Hewitt C. (2019). Hybrid life-cycle assessment for robust, best-practice carbon accounting. J. Cleaner Prod..

[cit39] Pan W., Li K., Teng Y. (2018). Rethinking system boundaries of the life cycle carbon emissions of buildings. Renew. Sustain. Energy Rev..

[cit40] Liu Z., Feng K., Hubacek K., Liang S., Anadón L. D., Zhang C., Guan D. (2015). Four system boundaries for carbon accounts. Ecol. Modell..

[cit41] Adewale C., Reganold J., Higgins S., Evans R. D., Carpenter-Boggs L. (2018). Improving carbon footprinting of agricultural systems: Boundaries, tiers, and organic farming. Environ. Impact Assess. Rev..

[cit42] Pasek A., Vaughan H., Starosielski N. (2023). The world wide web of carbon: Toward a relational footprinting of information and communications technology's climate impacts. Big Data Soc..

[cit43] Yue Z., Liu H., Xu Z., Wang Y. (2023). Evaluation of sustainability in northern Xinjiang based on ecological footprint-planetary boundary system framework. Ecol. Indic..

[cit44] Davis S. J., Peters G. P., Caldeira K. (2011). The supply chain of CO_2_ emissions. Proc. Natl. Acad. Sci. U. S. A..

[cit45] Jiang M., An H., Gao X., Zheng H., Li Y. (2021). Identifying the key sectors in the carbon emission flows along the production chain paths: A network perspective. Ecol. Indic..

[cit46] Wang H., Li L., Lin Z., Abulimiti A., Guan M., Zhao T., Tian Y. (2025). Optimization of Greenhouse Gas Accounting Methods for Wastewater Treatment Plants in East Chinese Regions: A Comparative Analysis of IPCC and Group Standards Based on 49 Plants in Shandong Province. Appl. Sci..

[cit47] Ding N., Xi Y., Jiang W., Li H., Su J., Yang S., Lie T. T. (2025). State-of-the-art carbon metering: Continuous emission monitoring systems for industrial applications. Heliyon.

[cit48] Saleem J., Khalid Baig Moghal Z., Tahir F., Al-Ansari T., Osman A. I., McKay G. (2025). Life cycle assessment of high value activated carbon production based on mass and functional performance metrics. Sci. Rep..

[cit49] Li N., Zhu Y.-W., Lin Y.-F., Yang Z., Chen G.-Q., Ma T.-M., Dai Q., Liu H.-Q. (2025). Life cycle sustainability assessment of activated carbon production technologies in China: Comparative analysis of coal-derived versus biomass-derived pathways. ACS Sustainable Chem. Eng..

[cit50] Vilén A., Laurell P., Vahala R. (2022). Comparative life cycle assessment of activated carbon production from various raw materials. J. Environ. Manage..

[cit51] Arena N., Lee J., Clift R. (2016). Life cycle assessment of activated carbon production from coconut shells. J. Cleaner Prod..

[cit52] Amin M., Shah H. H., Usman M., Iqbal M. A. (2025). The environmental impacts via life cycle assessment of activated carbon produced by Fiddlewood and Jacaranda biomass. Clean Technol. Environ. Policy.

[cit53] Alade A. O., Amuda O. S., Bello M. O. (2012). Life cycle inventory analysis (LCIA) of production of activated carbons from selected agricultural materials. Elixir Pollut..

[cit54] Oloriegbe T., Okofu C. A., Ibrahim F. B. (2023). The use of selected carbonized agricultural waste as a low cost scrubber in air pollution control. Rom. J. Transp. Infrastruct..

[cit55] Lee B., He J. (2025). Circular carbon capture: Comparative life cycle and techno-economic assessment of waste-derived activated carbons. Environ. Sci. Technol..

[cit56] Sherugar P., Padaki M., Naik N. S., George S., Murthy D. H. (2021). Biomass-derived versatile activated carbon removes both heavy metals and dye molecules from wastewater with near-unity efficiency: Mechanism and kinetics. Chemosphere.

[cit57] Queiroz L. S., De Souza L. K. C., Thomaz K. T., Lima E. T. L., Da Rocha Filho G. N., Nascimento L. D. A. D., De Oliveira Pires L. H., Faial K., Da Costa C. E. F. (2020). Activated carbon obtained from Amazonian biomass tailings (acai seed): Modification, characterization, and use for removal of metal ions from water. J. Environ. Manage..

[cit58] Clouse D. E., He J., Wang D., Davis V. A. (2025). Tailored activated carbons from biomass components for aqueous PFAS adsorption. Mater. Lett..

[cit59] Cruz Junior O., Campello I., Casco M. E. (2023). *et al.*, Enhanced CO_2_ capture by cupuassu shell-derived activated carbon with high microporous volume. Carbon Lett..

[cit60] Al-Ghurabi E. H., Boumaza M. M., Al-Masry W., Asif M. (2025). Optimizing the synthesis of nanoporous activated carbon from date-palm waste for enhanced CO_2_ capture. Sci. Rep..

[cit61] Ogawa M., Okimori Y., Takahashi F. (2006). Carbon sequestration by carbonization of biomass and forestation: Three case studies. Mitig. Adapt. Strateg. Glob. Change.

[cit62] Kacaribu A. A., Aisyah Y., Hidayat, Mawardiana F., Anwar K., Damanik M. R. F., Kemala P. (2026). Irfan and Darwin, Biomass-derived carbon materials as functional
soil interfaces for sustainable agriculture. Resour. Chem. Mater..

[cit63] Enebe M. C., Ray R. L., Griffin R. W. (2025). The impacts of biochar on carbon sequestration, soil processes, and microbial communities: A review. Biochar.

[cit64] Oyebiyi O. O., Laezza A., Hoque M. M., Thammavongsa S., Li M., Tsipas S., Tasiopoulos A. J., Scopa A., Drosos M. (2026). Organic amendments for sustainable agriculture: Effects on soil function, crop productivity and carbon sequestration under variable contexts. C.

[cit65] Niu J., Guan J., Dou M., Zhang Z., Kong J., Wang F. (2020). Sustainable synthesis of biomass-derived carbon electrodes with hybrid energy-storage behaviors for use in high-performance Na-ion capacitors. ACS Appl. Energy Mater..

[cit66] Wei L., Zeng J., Guo X. (2017). Bio-inspired high-performance solid-state supercapacitors with electrolyte, separator, binder and electrodes entirely from kelp. J. Mater. Chem. A.

[cit67] Masika E., Mokaya R. (2012). Hydrogen storage in high surface area carbons with identical surface areas but different pore sizes: Direct demonstration of the effects of pore size. J. Phys. Chem. C.

[cit68] Hwang S. H., Kim Y. K., Seo H. J., Jeong S. M., Kim J., Lim S. K. (2021). The enhanced hydrogen storage capacity of carbon fibers: The effect of hollow porous structure and surface modification. Nanomaterials.

[cit69] Ali A. H., Wanderlind E. H., Almerindo G. I. (2024). Activated carbon obtained from malt bagasse as a support in heterogeneous catalysis for biodiesel production. Renew. Energy..

[cit70] Ao S., Rashid U., Shi D., Rokhum S. L., Thuy L. T. T., Alahmadi T. A., Chinnathambi A., Mathimani T. (2024). Synthesis and utilization of biomass-derived sulfonated heterogeneous catalyst-BT-SO_3_H for microalgal biodiesel production. Environ. Res..

[cit71] Borand M. N., Çevik B., Bayrakdar Ateş E. (2025). Heterogeneous catalysts for biodiesel production: Development of bimetallic catalysts supported by activated carbon. J. Turk. Chem. Soc., Sect. B: Chem. Eng..

[cit72] He L., Zhang L.-L., Zhou H., Nie Y., Wang H., Tang B., Li H., Ma T., Zhang H. (2025). Boosting photocatalytic upcycling of liquid biomass into biodiesel via microenvironment modulation. Adv. Energy Mater..

[cit73] Mutushev A., Kaya A., Tulepov M., Kudyarova Z., Baiseitov D., Mukhanov D. (2025). Design and development of carbon–silicon-based air purification filters with antibacterial properties. Processes.

[cit74] Raso R. A., Zeltner M., Stark W. J. (2014). Indoor air purification using activated carbon adsorbers: Regeneration using catalytic combustion of intermediately stored VOC. Ind. Eng. Chem. Res..

[cit75] Zhou J., Wang L., Zhang P., Chen Y., Zhang L., Ma H., Zhang Y., Chen D. (2020). Features and commercial performance of a system of biomass gasification for simultaneous clean heating and activated carbon production. ACS Omega.

[cit76] de la Torre E., Redrovan A. S., Aragón-Tobar C. F. (2025). Progressive conversion model applied to the physical activation of activated carbon from palm kernel shells at the pilot scale in a Nichols furnace and at the industrial scale in a rotary kiln. Molecules.

[cit77] Karimi M., Shirzad M. (2024). Sustainable industrial process design for derived CO_2_ adsorbent
from municipal solid wastes: Scale-up, techno-economic and parametric assessment. Sustainable Mater. Technol..

